# Particle Collection in Imhoff Sedimentation Cones Enriches Both Motile Chemotactic and Particle-Attached Bacteria

**DOI:** 10.3389/fmicb.2021.643730

**Published:** 2021-04-01

**Authors:** Anneke Heins, Greta Reintjes, Rudolf I. Amann, Jens Harder

**Affiliations:** ^1^Department of Molecular Ecology, Max Planck Institute for Marine Microbiology, Bremen, Germany; ^2^Lethbridge Research and Development Centre, Agriculture and Agri-Food Canada, Lethbridge, AB, Canada

**Keywords:** phytoplankton, diatom bloom, particle-associated bacteria, Helgoland, microbial diversity, ecological succession, chemotaxis, phycosphere

## Abstract

Marine heterotrophic microorganisms remineralize about half of the annual primary production, with the microbiomes on and around algae and particles having a major contribution. These microbiomes specifically include free-living chemotactic and particle-attached bacteria, which are often difficult to analyze individually, as the standard method of size-selective filtration only gives access to particle-attached bacteria. In this study, we demonstrated that particle collection in Imhoff sedimentation cones enriches microbiomes that included free-living chemotactic bacteria and were distinct from particle microbiomes obtained by filtration or centrifugation. Coastal seawater was collected during North Sea phytoplankton spring blooms, and the microbiomes were investigated using 16S rRNA amplicon sequencing and fluorescence microscopy. Enrichment factors of individual operational taxonomic units (OTUs) were calculated for comparison of fractionated communities after separation with unfractionated seawater communities. Filtration resulted in a loss of cells and yielded particle fractions including bacterial aggregates, filaments, and large cells. Centrifugation had the lowest separation capacity. Particles with a sinking rate of >2.4 m day^–1^ were collected in sedimentation cones as a bottom fraction and enriched in free-living chemotactic bacteria, i.e., *Sulfitobacter*, *Pseudoalteromonas*, and *Vibrio*. Subfractions of these bottom fractions, obtained by centrifugation, showed enrichment of either free-living or particle-attached bacteria. We identified five distinct enrichment patterns across all separation techniques: mechano-sensitive and mechano-stable free-living bacteria and three groups of particle-attached bacteria. Simultaneous enrichment of particle-attached and chemotactic free-living bacteria in Imhoff sedimentation cones is a novel experimental access to these groups providing more insights into the diversity, structure, and function of particle-associated microbiomes, including members of the phycosphere.

## Introduction

Organic substrates for heterotrophic bacteria are unevenly distributed in the ocean. Particles, such as algal cells, transparent polymeric particles, marine snow, and fecal pellets, form temporary nutrient-rich hot spots in a large oligotrophic environment (Azam, 1998; Simon et al., 2002; Stocker, 2012). As a response, different heterotrophic lifestyles have evolved in this dynamic and heterogeneous habitat. These are (i) non-motile small bacteria with transporters adapted to low nutrient concentrations; (ii) motile bacteria, which sense nutrient gradients and swim toward food sources; and (iii) bacteria attached to and often gliding on particulate organic matter (Stocker, 2012; Yawata et al., 2014). The last two ecotypes are referred to as particle-associated bacteria, which are enriched in the interaction zone between bacterioplankton and particles. Non-living particulate organic matter drives the oceanic carbon pump and provides heterotrophic life below the photic zone with nutrients and energy until the particle is degraded (Azam, 1998). Living algae, in contrast, provide with their exudates a continuous substrate source for heterotrophic bacteria (Seymour et al., 2017). Bacteria in close proximity to the living algae are defined as the phycosphere microbiome and are currently understudied (Seymour et al., 2017).

Particle association is more difficult to track than physical attachment to particles. It is well established that particle-attached bacteria account for 0.1–4% of the whole bacterioplankton (Alldredge et al., 1986) but can reach higher local densities (Caron et al., 1982; Alldredge et al., 1986; Fernández-Gómez et al., 2013). Although they make up only a small fraction of the community, they have a disproportionally high impact on the biogeochemical element cycles, based on a significantly higher cellular metabolic activity (Smith et al., 1992; DeLong et al., 1993; Grossart et al., 2007; Ziervogel and Arnosti, 2008; Ziervogel et al., 2010). Particle-attached bacteria are larger than free-living bacteria, and their genomes encode additional degradative pathways (Caron et al., 1982; Alldredge et al., 1986; Simon et al., 2002; Smith et al., 2013; Rieck et al., 2015; Kappelmann et al., 2019). Motile bacteria also have enlarged genomes because they carry additional genes for chemotaxis and motility. They often represent ∼10% of cells in coastal seas (Stocker and Seymour, 2012), with large variations of up to 80% (Mitchell et al., 1995; Grossart et al., 2001).

The common technique to separate free-living and particle-attached bacteria is filtration (Bidle and Fletcher, 1995; Crump et al., 1999; Ayo et al., 2001; Crespo et al., 2013; Bižić-Ionescu et al., 2014), with delimiting filter pore sizes ranging from 0.8 (Phillips et al., 1999; Schapira et al., 2012) to 30 μm (Fuchsman et al., 2011). Recently, 3-μm filters have become the most frequently used pore size to target particle-attached bacteria in coastal waters (Eloe et al., 2011; Teeling et al., 2012, 2016; Crespo et al., 2013; D’Ambrosio et al., 2014; Chafee et al., 2018). The resulting size-selected communities are shaped not only by the filter pore size (Mestre et al., 2017) but also by filtration conditions. The retentate may decrease the effective pore size in the process of the filtration (Padilla et al., 2015). Mechanical stress was discussed to disintegrate fragile bacteria during filtration (Ferguson et al., 1984). Still, many taxa are well-separated in a 3–0.2 μm fraction often referred to as free-living and a > 3 μm particle-attached fraction. Most unicellular cyanobacteria, members of the SAR11 and SAR86 clades, and the flavobacterial marine group NS5 end up in the free-living fraction (Teeling et al., 2012, 2016; Mestre et al., 2020), whereas *Rhodobacterales*, *Alteromonadales*, *Bacteroidetes*, *Planctomycetes*, and Verrucomicrobia are enriched in particle fractions (Mestre et al., 2017). In considering this categorization, it is important to recall that particles have a limited life span (Iversen and Ploug, 2010). Hence, particle-attached bacteria must spend some time free-living on their way to the next particle (Bižić-Ionescu et al., 2014).

Scientists interested in the particle-associated microbiome must realize that filtration works on particle-attached bacteria but fails in the enrichment of the chemotactic free-living fraction. Motile genera, e.g., *Pseudoalteromonas*, *Colwellia*, *Shewanella*, and *Vibrio*, were detected in the expected size fraction for free-living bacteria, but also in particle fractions of larger size (Mestre et al., 2017). To resolve this separation problem, we explored Imhoff sedimentation cones as a separation tool to provide experimental access to the whole particle-associated microbiome. These cones were developed for quantifying settable particles in wastewater treatment plants (Novotny et al., 1989) but have not been used in marine microbiology. The steep incline in the cones results in the concentration of particles in a small volume at the bottom, which can be readily sampled. In this study, we compared fractionation in Imhoff cones with other separation techniques (filtration, plankton nets, and centrifugation) analyzing coastal seawater samples during diatom-dominated spring algal blooms at the long-term ecological research site Helgoland Roads. There, substrate-induced bacterial succession has been studied with an emphasis on the 3–0.2 μm fraction (Teeling et al., 2012, 2016; Chafee et al., 2018; Krüger et al., 2019). For comparing the separation technologies, we applied not only next-generation sequencing (NGS) of PCR-amplified partial 16S rRNA genes but also microscopic techniques. This study is a response to the call for exploration of alternative separation techniques for particle-attached bacteria made by Padilla et al. (2015).

## Materials and Methods

### Seawater and Plankton Net Sampling

**FIGURE 1:**
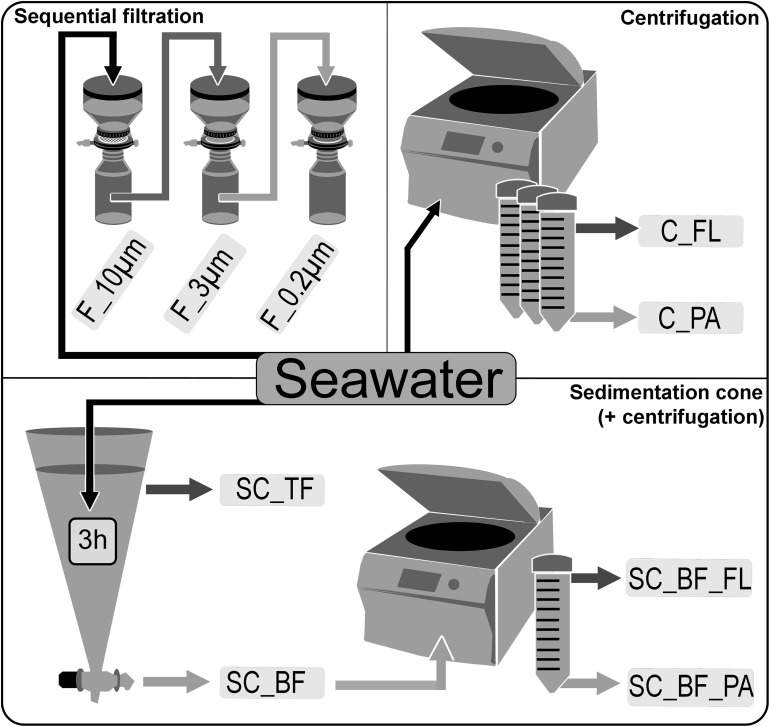
Overview of separation methods to fractionate seawater during a phytoplankton spring bloom in 2018 off Helgoland (North Sea). Size-delimiting separation was performed by sequentially filtration through 10-, 3-, and 0.2-μm filters (F_10μm, F_3μm, and F_0.2μm, respectively). Forced gravitational separation was achieved by centrifugation, resulting in a particle pellet (C_PA) and a supernatant with free-living cells (C_FL). Natural gravitational separation using Imhoff sedimentation cones used a 3-h settlement, resulting in a bottom fraction (SC_BF) and a top fraction (SC_TF). In a second separation, the bottom fraction (SC_BF) was divided by centrifugation into a supernatant with free-living cells (SC_BF_FL) and a pellet with particle-attached cells (SC_BF_PA).

Seawater was sampled at five time points (Supplementary Figure 1) in the Spring 2018 at the long-term ecological research site Helgoland Roads (54°11′03′′N, 7°54′00′′E). Samples were taken at about 1 m below the sea surface. The water was stored at 4°C in 10-L containers and processed within 2 h of sampling. Particles that had already settled during that time were resuspended by gentle inversion of the containers before the seawater was used for the experiments. The bacterial diversity of unfractionated seawater samples was compared with that of fractions obtained by sequential filtration, centrifugation, and sedimentation in Imhoff cones (Figure 1).

In this study, all filtrations were performed with a constant vacuum pressure of −200 mbar. Filters of all pore sizes had a diameter of 47 mm, an effective filter diameter of 39 mm, and an effective filter area of 1,195 mm^2^ and were composed of a hydrophilic polycarbonate membrane (Millipore, Darmstadt, Germany). All 0.2-μm pore-sized filters had the filter code GTTP. If during filtration a decrease in flow rate was detected, indicating a lower efficient pore size because of pore clogging, the filtration was repeated with a smaller sample volume.

For community analysis by 16S rRNA amplicon sequencing, 0.5 L of unfractionated seawater was filtered directly onto 0.2-μm pore-sized filters, frozen in liquid nitrogen, and stored at −80°C.

For cell staining by catalyzed reporter deposition fluorescence *in situ* hybridization (CARD-FISH) experiments and for determination of total cell counts, 50 ml of unfractionated seawater was fixed with 1% formaldehyde for 1 h at room temperature (RT) and filtered onto a 0.2-μm pore-sized filter. A cellulose acetate filter (0.45-μm pore size, Millipore, Darmstadt, Germany) supported all 0.2-μm filters used for cell counting to allow an even spread of the fixed cells. Filters for CARD-FISH were stored at −20°C.

In addition to the unfractionated seawater samples, plankton net samples were taken using 20- and 80-μm pore-sized nets. For DNA extraction and subsequent amplicon sequencing, 100 ml of these concentrated plankton net samples was filtered directly onto 3-μm filters.

### Size-Selective Separation by Sequential Filtration

Sequential filtration was used to fractionate seawater into large (>10 μm) and small (10–3 μm) particle fractions, as well as a free-living fraction (3–0.2 μm) (Figure 1). For community analysis by 16S rRNA amplicon sequencing, 1 L of seawater was first filtered through a 10-μm pore-sized filter (filter code of all 10-μm filters: TCTP), then the whole filtrate was applied to a 3-μm pore-sized filter (filter code of all 3-μm filters: TSTP), and finally, 0.5 L of the 3-μm filtrate was filtered through a 0.2-μm filter. For CARD-FISH, 200 ml of fixed seawater (2% formaldehyde, 2 h, RT) was applied to a 10-μm filter, then 200 ml of the 10-μm filtrate was filtrated through a 3-μm filter, and finally 50 ml of the 3-μm filtrate was used for the 0.2-μm filtration.

### Separation by Centrifugation

Forced settlement of particles was performed with 0.5 L of seawater in 50-ml tubes in a Rotina 35R centrifuge (Hettich, Tuttlingen, Germany), applying 4,890 × *g* for 10 min (Figure 1). Pellets in 1 ml of bottom water were collected using a sterile glass pipette and combined as the particle fraction of the centrifugation approach. The remaining 49 ml supernatant was collected and combined as the free-living fraction of the centrifugation approach. The particle fraction was resuspended in 5 ml of artificial seawater and filtered onto a 0.2-μm filter for later DNA extraction. For CARD-FISH experiments, 1 ml of sample was fixed (2% formaldehyde, 2 h, RT), diluted 10-fold with artificial seawater, and then filtered onto a 0.2-μm pore-sized filter. The free-living fraction was filtered directly through a 0.2-μm filter for amplicon sequencing, or a 10-ml portion was fixed as aforementioned and then filtered onto a 0.2-μm filter for CARD-FISH experiments.

### Separation in Sedimentation Cones

Imhoff cones were applied in this study to fractionate microorganisms in seawater by natural gravitation (Figure 1). In a preliminary study in Spring 2017, on Julian Days 88, 94, and 135, 1 L of seawater was sampled and directly filtered through a 3-μm filter and then onto a 0.2-μm pore-sized filter. In sedimentation cones, 1 L of seawater was allowed to settle for 24 h. A bottom fraction of 1 to 5 ml was withdrawn using the bottom stopcock (Figure 1). The bottom fraction was filtered onto 3-μm pore-sized filters. DNA was extracted as described in Zhou et al. (1996). The 16S rRNA gene amplicon sequencing and analysis were performed as described below.

The preliminary study indicated the possibility of a bottle effect on the community composition. Hence, in 2018, different sedimentation times (0.5, 1, 3, 6, 16, and 24 h) were tested to find optimal conditions for the spring bloom samples. Large particles that settled within 1 h were rare, whereas particles visible to the naked eye had settled after 3 h. Samples collected after 24 h of sedimentation were analyzed to explore the effects of long settlement times. A 3-h sedimentation was chosen as standard throughout the experiments.

Bottom fractions containing settled particles and associated bacteria in 1- to 5-ml volumes were combined from several cones. Of this mixed sample, 5 ml was filtered onto a 0.2-μm filter for DNA extraction. One milliliter was fixed (2% formaldehyde, 2 h, RT), diluted 10-fold in artificial seawater, and filtered onto a 0.2-μm filter for cell staining. For the free-living fraction, 0.5 L of the remaining water of one cone (top fraction) was filtered onto a 0.2-μm filter, which was later used for DNA extraction. Ten milliliter was fixed as aforementioned and then filtered onto a 0.2-μm filter for cell staining.

A further separation of the bottom fraction from the Imhoff cones was performed by centrifugation. For this, the bottom fraction of one sedimentation cone was suspended in 50 ml of artificial seawater and then centrifuged (4,890 × *g* for 10 min). The obtained pellet was expected to contain particle-attached bacteria, and the supernatant was expected to contain free-living bacteria, including motile chemotactic bacteria. The particle fraction of this separation consisted of all particles in 5 ml bottom water and was filtered directly onto a 0.2-μm filter for DNA extraction, or 1 ml was fixed (as aforementioned) and diluted 10-fold in artificial seawater prior to filtration onto a 0.2-μm filter. The supernatant was directly filtered for DNA extraction (0.2-μm filter), and 10 ml was fixed as aforementioned and then filtered (0.2-μm filter) for cell staining.

### 16S rRNA Gene Sequencing and Analysis

DNA of all 2018 samples was extracted from filters using the Power Water Kit according to the manufacturer’s protocol (Qiagen, Hilden, Germany). DNA concentrations and fragment lengths were analyzed in a capillary electrophoresis (Fragment Analyzer, Advanced Analytical, Santa Clara, United States) using the DNF-488 High Sensitivity Genomic DNA Analysis Kit (Advanced Analytical). One nanogram of DNA served as template for a 25 μl PCR with 2.5 U Taq DNA polymerase (Merck, Darmstadt, Germany), 20 mM of Tris pH 8.4, 50 mM of KCl, 0.6 mM of MgCl_2_, 90 μM of bovine serum albumin, 0.0625 mM of each dNTP, 0.4 μM of barcoded forward primer Bakt_341F (5′-TATACGCCTACGGGNGGCWGCAG-3′) and 0.4 μM of reverse primer Bakt_805R (5′-GACTACHVGGGTATCTAATCC-3′). The primer pair targets the hypervariable V3 and V4 regions of the bacterial 16S rRNA gene (Herlemann et al., 2011). The amplification program involved initial denaturation for 3 min at 94°C, 30 cycles of denaturation for 45 s at 94°C, annealing for 1 min at 50°C, elongation for 1.5 min at 72°C, and a final elongation of 10 min at 72°C. Amplicons were purified using AMPure XP beads (Beckman Coulter, Krefeld) and were quantified with the DNF-473 Standard Sensitivity NGS Fragment Analysis Kit (1–6,000 bp, Advanced Analytical) on the Fragment Analyzer. Equimolar amplicon pools were sequenced on an Illumina HiSeq2500 in rapid mode with 2 × 250 bp paired-end run performed by the Max Planck Genome Centre Cologne, Germany^1^.

A total of 19.8 million 16S rRNA reads were paired with BBmerge v37.82 applying default settings and allowing no mismatch in the overlapping region (Bushnell et al., 2017). A total of 12.9 million merged reads were then de-multiplexed and quality trimmed using MOTHUR (Schloss et al., 2009). The command “trim.seqs” was used with the setting of a minlength = 300, maxambig = 0, maxhomop = 8, allfiles = T, and checkorient = T. The final high-quality merged sequences (8.9 million) were classified with the SILVAngs pipeline (Quast et al., 2013) using SILVA release version 132. We used a minimum alignment length of 150, a minimum quality score of 30 (minimum length of a sequence/reads), and a similarity threshold of 0.98 for the creation of operational taxonomic units (OTUs). Each OTU was classified according to its most related genus. The relative read abundance of sequences affiliating to chloroplasts, mitochondria, Eukarya, or Archaea were determined for each sample. After their proportional quantification, they were removed to focus the dissimilarity analysis on bacterial reads (Supplementary Figure 2). Read abundance was normalized for each sample using the “decostand()” function (method = total) (Oksanen et al., 2020). A minimum of 26,231 and maximum of 312,329 reads per sample were analyzed after processing. Raw reads of all samples were deposited at the European Nucleotide Archive (ENA) and are accessible through the accession number PRJEB41742.

An enrichment factor was calculated by dividing the relative read abundance of an OTU in a fraction by the relative read abundance of the OTU in unfractionated seawater at the respective time point. For example, the relative read abundance of SAR 11 clade Ia varied in unfractionated seawater samples across all five time points (min–max: 2.3–18.9%) but was always higher in the filter-fractionated free-living samples (min–max: 21.7–39.3%) and lower in the large filter-fractionated particle fractions (min–max: 0.37–1.6%). This led to an average enrichment factor of 4.15 for the filter-fractionated free-living and an enrichment factor of 0.10 for the large particle fraction, indicating enrichment (EF > 1) and depletion (EF < 1), respectively.

### Cell Quantification

CARD-FISH filters were processed following the protocol of Pernthaler et al. (2004) with four modifications. (i) The agarose (0.1% w/v, LE agarose, Biozym, Hessisch Oldendorf, Germany) embedding of the filters was done on parafilm instead of glass to avoid particle loss: a drop of agarose was applied to the parafilm. The filter was carefully dipped into the drop from both sides before being placed with the filtered side facing down on the parafilm. (ii) After the lysozyme treatment, filters were incubated with 60 U of achromopeptidase (Sigma, Taufkirchen, Germany) in 10 mM of NaCl and 10 mM of Tris HCl pH 8.0 for 30 min at 37°C to permeabilize the cell walls. Then filters were rinsed with MilliQ water. (iii) Endogenous peroxidases were inactivated with 0.15% hydrogen peroxide in methanol for 20 min at RT. (iv) Finally, filters were embedded in a 4:1 ratio of Citifluor to VectaShield plus 4′,6-diamidino-2-phenylindole (DAPI; 0.5 ng L^–1^) as described in Nikrad et al. (2012).

Cells were manually counted with a Zeiss Axio Imager D2 microscope (Zeiss, Oberkochen, Germany) equipped with a Zeiss AxioCam MRm camera, utilizing the software Axio Vision (v 4.8). Detection of cells was based on fluorescence signals of DAPI using the microscope filter DAPI HC (AHF, Tübingen, Germany) and of Alexa 488 using the filter EGFP ET (AHF). The latter was used in CARD-FISH for the probes EUBI-III and Non-EUB (control) (Amann et al., 1990). Per sample, a minimum of 2,000 cells or the total cell abundance in 100 grids (size of one grid: 15,625 μm^2^) were counted. The local density of cells on the filters was analyzed to determine the spatial proximity to a particle and other cells. We differentiated seven types of cell distribution: (1) single cells with equal spreading across the grid; (2) cells in direct contact with a transparent exopolymer particle (TEP); (3) an algae; (4) diatom shells; (5) cell consortia with a larger local cell density compared with the surrounding background cell density, but without stained contact points; (6) cells in filaments of at least three cells; and (7) cells in aggregates with a central distinctive connection among them (Supplementary Figure 3). The total cell count per milliliter of seawater was calculated with the following equation:

$$\frac{cells}{\text{ml}}=\frac{totalcells}{countedgrids}\times \frac{effectivefilterarea\left({\text{m}}^{2}\right)}{countinggridarea\left({\text{m}}^{2}\right)}$$

$$\times \frac{1}{filteredvolume\left(\text{ml}\right)}$$

Particles with cells were analyzed for their abundance and approximate size. Based on their appearance, they were categorized as transparent polymeric particles, algae, or diatom debris.

### Statistical Evaluation and Graphical Visualization

Statistical analyses, data transformations, and graphing were performed in R studio (R Core Team, 2014). Non-metric multidimensional scaling (NMDS) analysis based on Bray–Curtis dissimilarity indices was performed with the “vegdist()” function (“vegan” package; Oksanen et al., 2020). Stress was determined with the “metaMDS()” function, and a Shepard diagram was created to determine the fit between observed dissimilarity against the ordination distance (non-metric and linear). Statistical evaluation of the communities between the methods was performed with a permutational multivariate analysis of variance (PERMANOVA) using distance matrices [method = bray, “vegdist()” function, “vegan” package; Oksanen et al., 2020]. This analysis was followed by a pairwise comparison of the method effect [“pairwise.perm.manova()” function, method = euclidian].

Cell distributions between different particle types, the colonization of cells and varying particle types, and the calculated diversity indices of each sample group were treated as comparisons between parametric groups. Significant differences among the defined parameters were determined with an analysis of variance [“aov()” function], followed by a Tukey multiple pairwise comparison [“TukeyHSD()” function] *t*-test. Residuals versus fit plots were used to check for homogeneity of variances, which was additionally tested by a Levene test [“leveneTest()” function, “car” package, R Version 3.5.3; Fox and Weisberg, 2019]. A normality plot of residuals was used to test for the normal distribution of the data. This was additionally tested by a Shapiro–Wilk test [“shapiro.test()” function] after extraction of the residuals [“residuals()” function]. If the data distribution did not meet the homogeneity of variance or violated a normality distribution, the one-way ANOVA was replaced by a non-parametric Kruskal–Wallis rank sum test. The rank sum test was followed by pairwise comparisons using Tukey and Kramer (Nemenyi) test with Tukey distance approximation for independent samples [posthoc.kruskal.nemenyi.test (dist = “Tukey”) function, “PMCMR” package, R Version 3.5.3; Pohlert, 2014].

Further R packages were used for data visualization and data transformation: ComplexHeatmap (Gu et al., 2016), circlize (Gu et al., 2014), picante (Kembel et al., 2010), rioja (Juggins, 2020), colorspace (Zeileis et al., 2009), and dplyr (Wickham et al., 2018).

## Results

**FIGURE 2:**
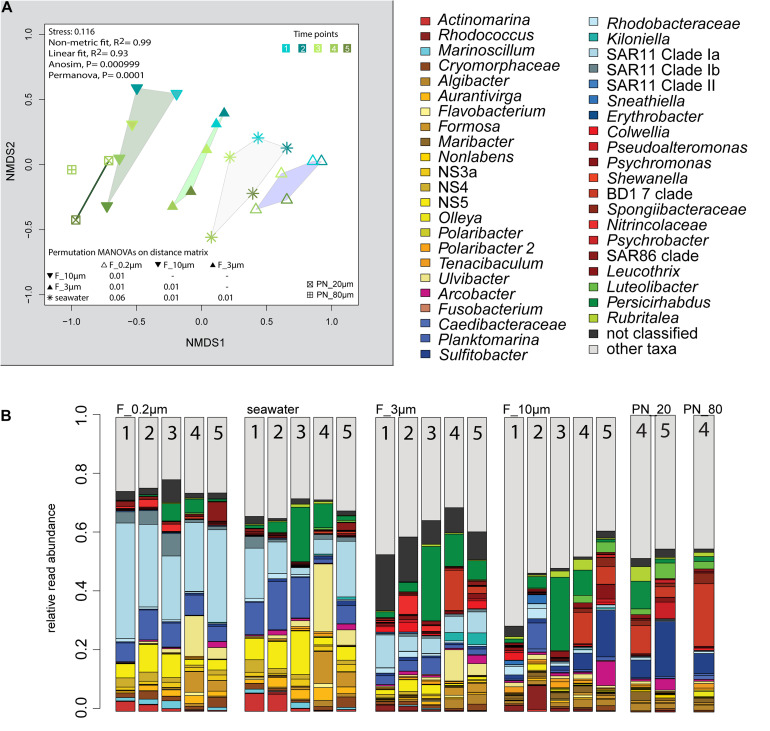
Bacterial community diversity of unfractionated seawater and size-fractionated populations obtained during a phytoplankton spring bloom off Helgoland (North Sea) in 2018. **(A)** Non-metric multidimensional scaling (NMDS) plot based on Bray–Curtis dissimilarity shows shifts in bacterial communities at five sampling time points (Julian Days 102, 109, 115, 128, and 142). The statistical evaluation is represented by an ANOVA, PERMANOVA, and a pairwise comparison visualized in a distance matrix (n for each group: 5). **(B)** Stacked bar charts of community composition. Operational taxonomic units (OTUs) shown were among the five most abundant OTUs in at least one sample. Time points are indicated as numbers inside the bars. *F_0.2μm*, filtered fraction ranging from 3 to 0.2 μm; *F_3μm*, filtered fraction ranging from 10 to 3 μm; *F_10μm*, filtered fraction >10 μm.

The 2018 phytoplankton spring bloom was dominated by diatoms of the genera *Thalassiosira*, *Chaetoceros*, *Mediopyxis*, and *Rhizosolenia* and the haptophyte *Phaeocystis*. The phytoplankton bloom was accompanied by an increase of the total bacterial cell counts from 0.60 × 10^6^ to 2.47 × 10^6^ cells ml^–1^ (Supplementary Figure 1 and Supplementary Table 1). Community dissimilarity analysis of unfractionated seawater samples showed a change with the onset of the algal bloom (Figure 2A). Dominant OTUs in the seawater were affiliated with *Planktomarina*; SAR11 clades Ia and Ib; *Ca.* Actinomarina; flavobacterial *Ulvibacter*, *Formosa*, and *Aurantivirga*; marine clades NS3a, 4, and 5; and *Persicirhabdus* of the Verrucomicrobia (Figure 2B).

### Sequential Filtration as Standard for Size-Selective Separation of Microbial Communities

**FIGURE 3:**
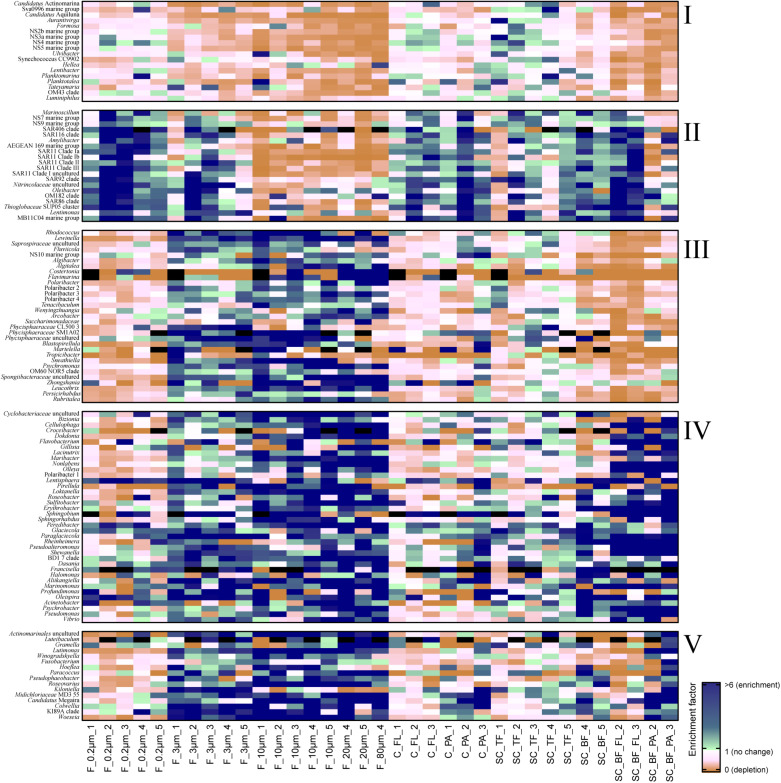
Heatmap of enrichment factors: experimental separation of cells resulted in five operational taxonomic unit (OTU) patterns that were characterized by **(I)** an overall depletion of free-living genera, **(II)** enriched free-living genera, **(III)** particle-attached genera that were not enriched in the bottom fraction, **(IV)** particle-attached genera present in the bottom fraction as free-living and as particle-attached cells, and **(V)** particle-attached genera enriched in the bottom fraction. *C_FL*, supernatant fraction of a separation by centrifugation (with free-living cells); *C_PA*, particle fraction of a separation by centrifugation; *F_0.2μm*, filtered fraction ranging from 3 to 0.2 μm; *F_3μm*, filtered fraction ranging from 10 to 3 μm; *F_10μm*, retentate fraction > 10 μm; *F_20μm*, plankton net catch > 20 μm; *F_80μm*, plankton net fraction >80 μm; *SC_TF*, sedimentation cone top fraction; *SC_BF*, sedimentation cone bottom fraction; *SC_BF_FL*, free-living cells in sedimentation cone bottom fraction; *SC_BF_PA*, particle-attached cells in sedimentation cone bottom fraction. Numbers at the end of the sample names indicate sampling time points (Julian Days 102, 109, 115, 128, and 142). *Dark blue*, enrichment factor ≥6; *light green*, enrichment factor 2–6; *white*, enrichment factor 1 (=no enrichment or depletion); *brown*, enrichment factor <1 (=depletion); *black*, OTU relative read abundance was in seawater below the detection limit, and thus, no enrichment factor could be calculated.

**FIGURE 4:**
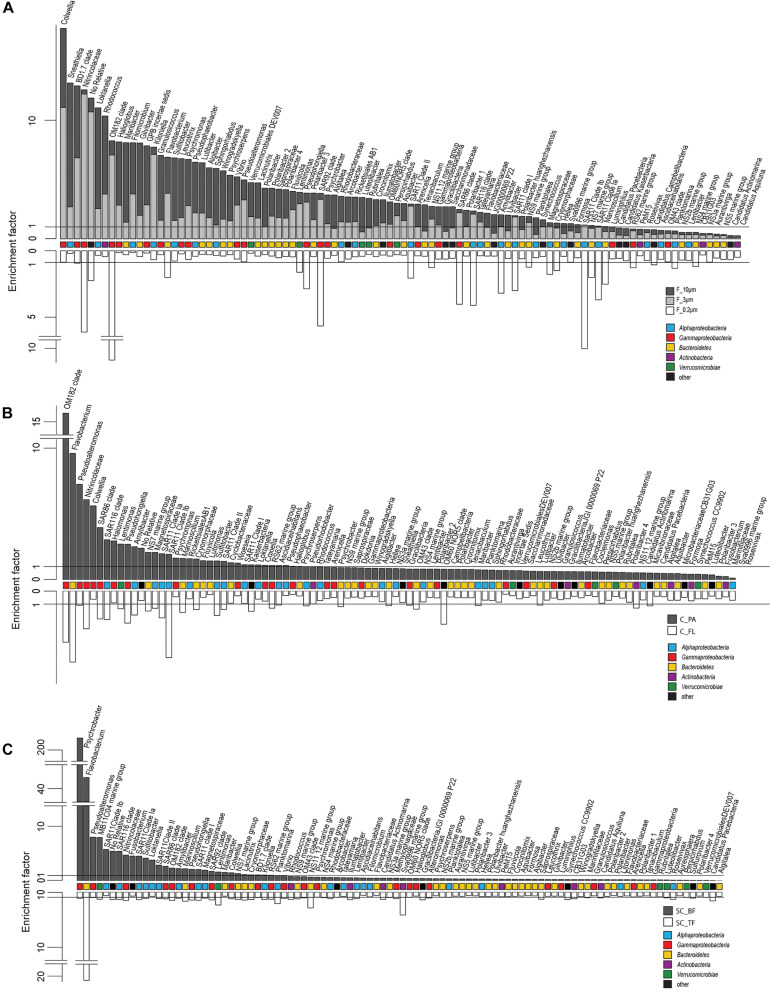
Two-sided bar charts of operational taxonomic units (OTUs) with, on average, the highest relative read abundance in seawater and sorted by their mean enrichment factor across time points in **(A)** the filtration-derived particle fraction (F_10μm and F_3μm), **(B)** centrifugation-derived particle fraction (C_PA), and **(C)** sedimentation cone derived bottom fraction (SC_BF) (*gray* and *dark gray*). Samples were obtained during a phytoplankton spring bloom off Helgoland (North Sea) in 2018. *White*, free-living (C_FL/F_0.2μm) and top fraction (SC_TF).

Microscopy of the 10-, 3-, and 0.2-μm pore-sized filters confirmed the effectivity of filtration. Particles including diatoms and *Phaeocystis* cells were collected on the 10- and 3-μm filters, whereas the 3–0.2 μm fraction contained almost exclusively single bacterial cells (98.3–100%) (Table 1). Only 44–71% of the cells present in unfractionated seawater were recovered on 0.2-μm filters after sequential filtration, with less than 1% of all cells detected on the 10- and 3-μm filters (Table 1). The cell loss coincided with shifts in OTU relative read abundances. To quantify the abundance shifts, we derived enrichment factors that were calculated as ratio of the relative read abundance in a fraction community divided by the relative read abundance in the unfractionated seawater community (Figure 3). We also calculated the mean enrichment factor for the OTUs with, on average, the highest relative read abundance in seawater (Figure 4).

**TABLE 1:** Total cell counts (TCC) of seawater fractions, collected during a phytoplankton spring bloom off Helgoland (North Sea) in 2018 (Julian Days 102, 109, 115, 128, and 142).

	Fraction	TCC (×10^3^ cells ml^–1^)			
		Mean	SD	FL^a^	PA^b^
TP1	C_FL	382.6	58.0	382.4	0.2
	C_PA	13.1	2.3	12.9	0.2
	F_0.2μm	438.3	21.9	431.0	7.4
	F_3μm	1.6	0.7	0.7	0.9
	F_10μm	1.1	0.6	0.4	0.7
	SC_TF	562.2	78.7	551.8	10.4
	SC_BF	1.5	0.2	1.5	0.0
	SC_BF_FL	1.5	0.5	1.4	0.1
	SC_BF_PA	0.3	0.0	0.3	0.0
TP2	C_FL	597.8	102.4	595.5	2.3
	C_PA	11.1	6.2	10.5	0.6
	F_0.2μm	469.0	52.4	464.1	4.9
	F_3μm	1.5	1.1	0.5	1.0
	F_10μm	1.4	2.0	0.1	1.3
	SC_TF	661.2	120.5	656.6	4.6
	SC_BF	1.1	0.8	1.0	0.1
TP3	C_FL	807.7	113.0	800.4	7.3
	C_PA	20.9	5.4	20.5	0.4
	F_0.2μm	738.4	61.6	738.4	0.0
	F_3μm	1.4	0.9	0.5	0.9
	F_10μm	1.6	2.2	0.2	1.5
	SC_TF	777.5	93.8	748.8	28.8
	SC_BF	0.4	0.2	0.4	0.0
	SC_BF_FL	0.3	0.2	0.3	0.0
	SC_BF_PA	0.1	0.1	0.0	0.1
TP4	F_0.2μm	922.4	94.4	916.2	6.3
	F_3μm	1.4	0.3	0.9	0.4
	F_10μm	1.6	2.0	0.8	0.9
	SC_TF	1,011.1	152.5	997.2	13.9
	SC_BF	1.3	0.4	1.2	0.0
TP5	F_0.2μm	1,677.9	191.6	1,655.8	22.1
	F_3μm	4.5	1.2	2.8	1.6
	F_10μm	5.8	6.1	2.4	3.4
	SC_TF	1,766.7	202.8	1,766.7	0.0
	SC_BF	3.5	0.7	2.2	1.3

*^a^Free-living cells (FL) and ^b^particle-attached cells (PA) were quantified by microscopy. C_FL/PA, centrifugation-derived free-living (= supernatant) and particle-attached (= pellet) fraction; F_0.2μm, filtered free-living fraction 3–0.2 μm; F_3μm, filtered particle fraction 10–3 μm; F_10μm, filtered particle fraction >10 μm; SC_BF, sedimentation cone bottom fraction after 3 h of sedimentation, resuspended and separated by centrifugation into free-living (SC_BF_FL) and particle-attached (SC_BF_PA) fractions; SC_TF, sedimentation cone free-living top fraction after 3 h of sedimentation; SD, standard deviation; TP, sampling time points.*

The 3–0.2 μm fractions were enriched in reads affiliated with SAR11 and depleted in several flavobacterial clades, notably *Aurantivirga* and *Formosa* (Figure 3). The fact that the latter was also depleted in the particle fractions (>10 and 10–3 μm) suggested cell loss of *Flavobacteriales*. The unfractionated seawater communities and derived free-living communities resembled each other closely, which was supported by pairwise statistical comparisons (Figure 2A and Supplementary Table 2), albeit with a lower diversity (Shannon–Wiener index) after filtration because of a lower evenness in relation to seawater communities (ANOVA, *P* < 0.001, Supplementary Figure 4).

Particle filters (>10 and 10–3 μm) were dominated by particle-attached bacteria. On average, less than 40% were single cells (Table 1). These cells were smaller than 3 μm based on DAPI staining. Most cells were in direct contact with transparent polymeric particles, algae, or diatom shells (Supplementary Figure 3). A small portion of cells appeared clustered together in cell consortia larger than 3 μm (Supplementary Table 3). Bacterial cell density on particles was highly variable. In general, transparent polymeric particles were slightly more populated [0.02 ± 0.02 cells (μm^2^ particle) ^–1^] than algae [0.01 ± 0.01 cells (μm^2^ particle) ^–1^] and diatom shells [0.01 ± 0.01 cells (μm^2^ particle) ^–1^] (Supplementary Figure 5).

The >10 and 10–3 μm fractions were significantly different from each other and from unfractionated seawater and the 3–0.2 μm fractions (ANOSIM and PERMANOVA, *P* < 0.001; Figure 2A and Supplementary Table 2). The diversity of the filtration-separated particle communities was higher than in unfractionated seawater and free-living communities because of greater OTU evenness (ANOVA, *P* < 0.001) (Supplementary Figure 4).

Only one of the dominant OTUs in seawater (Figure 2B and Supplementary Table 4) had a higher relative read abundance in the filtered particle fractions: *Persicirhabdus* (Verrucomicrobia). The other dominant seawater OTUs were depleted.

Operational taxonomic units with a high relative read abundance in seawater are, for arithmetical reasons, unlikely to have high enrichment factors. For OTUs with a relative read abundance of at least 0.1% in seawater, only few OTUs had enrichment factors >6 in a particle fraction. These were expected to spend most of their lifetime on particles. Examples of these OTUs in both particle fractions were in decreasing order of the read abundance: clade BD1-7, *Sulfitobacter*, *Algibacter*, *Rhodococcus*, *Colwellia*, *Psychromonas*, *Winogradskyella*, and *Maribacter* (Figure 3). A size selectivity was noticed for several OTUs: uncultured Nitrincolaceae (*Oceanospirillaceae*), *Kiloniella*, and *Phycisphaeraceae* were present at higher relative read abundances in the small particle communities (10–3 μm), whereas the large particle communities (>10 μm) included higher relative abundances of *Sneathiella*, *Loktanella*, and *Maribacter*. The taxa with a lower relative read abundance in seawater (<0.1%) and with high enrichment (enrichment factors of >25) in both particle communities were *Tropicibacter*, *Lentisphaera*, *Roseobacter*, and uncultured Actinomarinales. Highly enriched on particle filters but less rare in seawater were uncultured *Cyclobacterium*, clade KI89A, *Paraglaciecola*, and *Phycisphaeraceae*.

We extended the size-selective separation by community analysis to 20- and 80-μm pore-sized plankton net catches that were concentrated on 3-μm filters. These fractions were similar to the corresponding >10-μm communities (Figure 2). Interestingly, *Gramella* was 125 times more enriched in plankton net samples than in unfractionated seawater and >10-μm samples (Figure 3 and Supplementary Table 4). Also highly enriched were *Aliikangiella*, *Costertonia*, *Gillisia*, and *Flavimarina*.

### Centrifugation as Forced Gravitational Separation of Microbial Communities

**FIGURE 5:**
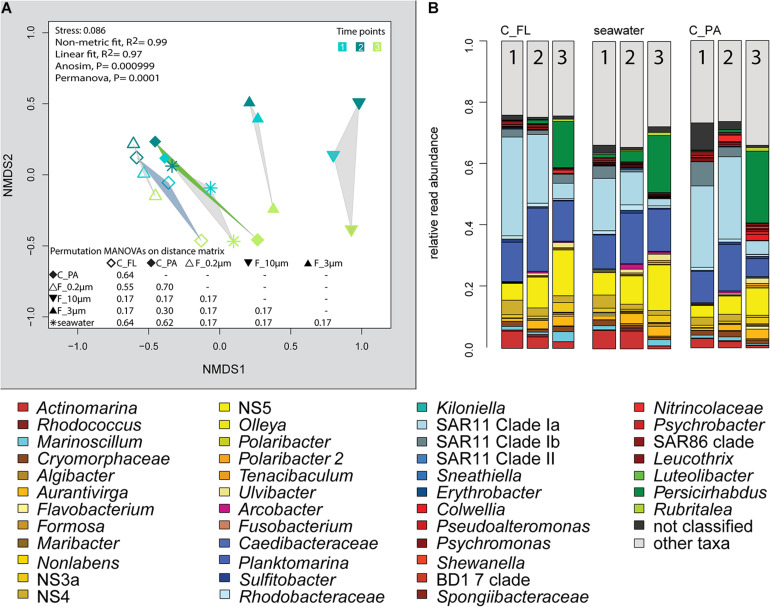
Bacterial community diversity of unfractionated seawater, density-, and size-fractionated populations obtained during a phytoplankton spring bloom off Helgoland (North Sea) in 2018. **(A)** Non-metric multidimensional scaling (NMDS) plot based on Bray–Curtis dissimilarity shows shifts in bacterial communities at three sampling time points (Julian Days 102, 109, and 115). The statistical evaluation is represented by an ANOVA, PERMANOVA, and a pairwise comparison visualized in a distance matrix (n for each group: 3). **(B)** Stacked bar charts of community composition. Operational taxonomic units (OTUs) shown were among the five most abundant OTUs in at least one sample. *C_FL*, supernatant fraction of a separation by centrifugation (with free-living cells); *C_PA*, particle fraction of a separation by centrifugation.

Centrifugation of seawater at 4,890 × *g* for 10 min resulted in visible pellets. Although the relative read abundance of 16S rRNA genes originating from chloroplasts increased in two out of three samples by 50% (Supplementary Figure 2), the bacterial communities of supernatant and pellet showed no significant differences (pairwise comparison, *P* > 0.05, Figure 5A and Supplementary Table 5). Diversity, species richness, and evenness were not significantly different between the fractions (Supplementary Figure 4). The particle fraction had more cells than the particle fractions of other methods (Table 1). Dominant OTUs had similar relative read abundances in seawater and in the pellet and supernatant communities (Figure 5B). Among the OTUs enriched in pellet fractions were *Profundimonas* and *Colwellia*, which had the highest read abundance of 1.9% at time point 3 (onset of the diatom bloom). OTUs with a relative read abundance of ≥ 0.1% and an enrichment factor of above 18 for the pellet fraction were *Ca.* Megaira and *Martelella* (both *Alphaproteobacteria*), *Aliikangiella*, *Oleispira*, *Profundimonas*, and *Pseudoalteromonas* (all *Gammaproteobacteria*), *Flavobacterium*, flavobacterial clade NS10, and *Lentisphaera* (Figure 3).

### Community Shifts by Prolonged Settlement Times in Imhoff Sedimentation Cones

Enrichment of particles in sedimentation cones was first explored at Helgoland Roads in Spring 2017 using a particle settlement time of 24 h. Particle communities were collected onto 3-μm pore-sized filters and were compared with seawater communities directly sampled as >3 and 3–0.2 μm fractions by sequential filtration. Statistical analyses revealed significant differences between the communities, with the largest distances between the 24-h particle samples and both types of 0-h samples (ANOVA and PERMANOVA, *P* < 0.001, Supplementary Tables 6, 7 and Supplementary Figure 6). Dominant free-living microorganisms (3–0.2 μm fraction) were affiliated to SAR11 clades, SAR86, SAR116, OM43, *Planktomarina*, *Ca*. Actinomarina, flavobacterial marine groups NS4 and NS5, and uncultured *Cryomorphaceae*, a typical free-living community for a pre-algal bloom in March at Helgoland Roads (Chafee et al., 2018) (Supplementary Table 8). At the earlier sampling days (time points 1 and 2, 2017), few particles were collected. The seawater >3-μm population showed increased relative read abundances of *Fluviicola* and the gammaproteobacterial genera *Colwellia*, *Halomonas*, *Pseudoalteromonas*, *Psychrobacter*, *Shewanella*, *Vibrio*, and *Woeseia*. Most of these gammaproteobacterial OTUs dominated the sedimentation cone bottom fraction (>3 μm), together with increased numbers of *Alphaproteobacteria* (*Erythrobacter*, *Loktanella*, *Sulfitobacter*, and *Roseovarius*) and the *Flavobacteriaceae Gramella* and *Olleya*. Many of these taxa are known to be part of the phycosphere but are also part of the “bottle effect,” a rapid shift in community composition after sampling in bottles (Pernthaler and Amann, 2005).

**FIGURE 6:**
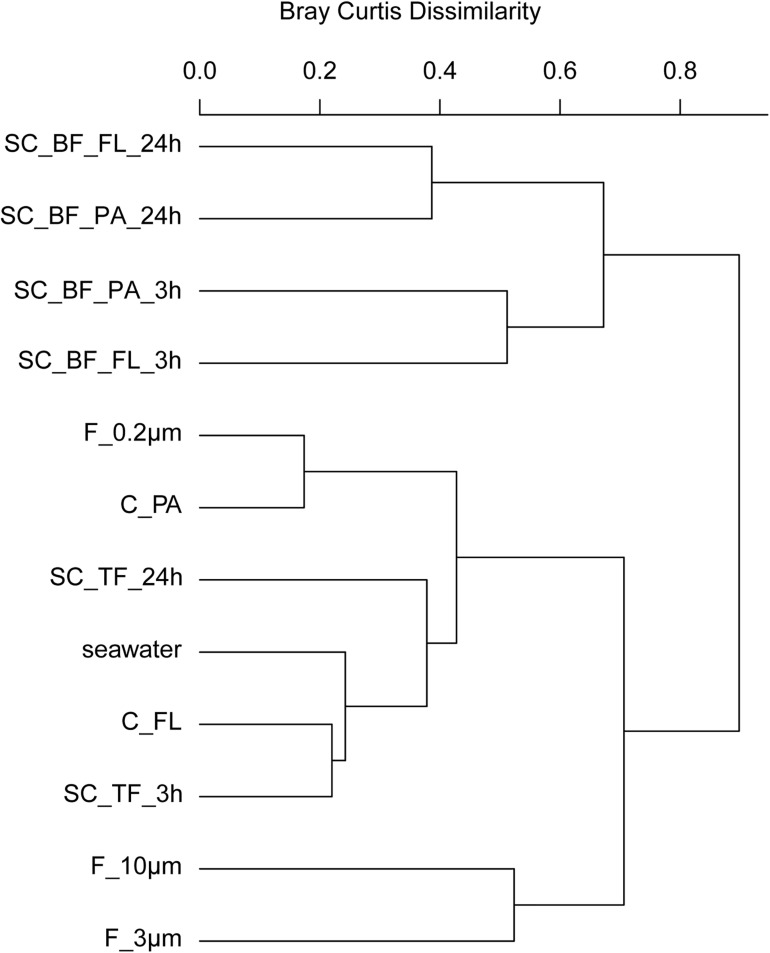
Community composition shifts by variable settlement times (3 and 24 h). Cluster tree based on Bray–Curtis dissimilarity depicting bacterial communities obtained during a phytoplankton spring bloom off Helgoland (North Sea) in 2018. Seawater was fractionated applying sequential filtration with 10-, 3-, and 0.2-μm pore-sized filters (F_10μm, F_3μm, and F_0.2μm, respectively), using centrifugation to obtain a supernatant (C_FL) and pellet fraction (C_PA) or by sedimentation in cones (SC). The SC samples were composed of a free-living fraction (SC_TF), and a bottom fraction that was resuspended in artificial seawater and secondarily split by centrifugation into a supernatant (SC_BF_FL) and pellet (SC_BF_PA). Sedimentation times were either 3 or 24 h. All other samples were immediately processed (*t* = 0 h). A reference sample was taken by filtering seawater directly onto a 0.2-μm filter (seawater).

In 2018, bacterial communities after 3 and 24 h of separation in cones were analyzed to quantify the influence of microbial growth. Bottom fraction communities of the two sedimentation times resembled each other closer than other communities (Figure 6). Growth of gammaproteobacterial *Pseudoalteromonas*, *Colwellia*, and *Psychrobacter* was detected as larger relative read abundance or enrichment factor in the 24-h sedimentation cone bottom fractions, complemented by lower relative read abundances and enrichment factors of *Olleya*, Polaribacter 1, *Pseudofulvibacter*, *Flavirhabdus*, uncultured Nitrincolaceae, *Dokdonia*, *Sulfitobacter*, *Roseobacter*, *Nonlabens*, and *Cellulophaga*. Such a strong growth-dependent signal was not observed in the supernatant fractions of the cones (Supplementary Tables 4, 8).

### Optimized Separation in Imhoff Sedimentation Cones

In Spring 2018, six settlement times (0.5, 1, 3, 6, 16, and 24 h) covering sinking speeds ranging from 0.3 m day^–1^ (24-h settlement time) to 14.4 m day^–1^ (0.5-h settlement time) were tested. The amount of particles settled at the bottom of the cones did not visibly increase beyond 3 h. This time corresponds to the collection of all particles with a sinking speed larger than 2.4 m day^–1^ and provides sufficient time for chemosensory-directed swimming of motile bacteria to follow the particles to the bottom of the cone (reviewed in Stocker and Seymour, 2012).

**FIGURE 7:**
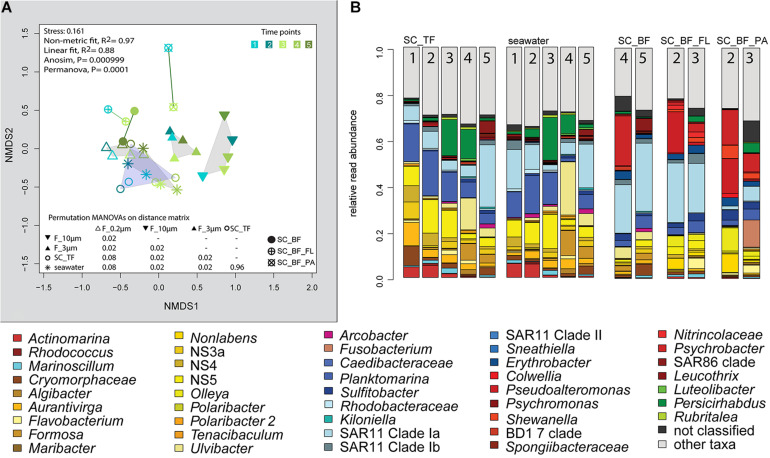
Bacterial community diversity of unfractionated seawater and gravitationally fractionated populations obtained during a phytoplankton spring bloom off Helgoland (North Sea) in 2018. **(A)** Non-metric multidimensional scaling (NMDS) plot based on Bray–Curtis dissimilarity shows shifts in bacterial communities at five sampling time points (Julian Days 102, 109, 115, 128, and 142). The statistical evaluation is represented by an ANOVA, PERMANOVA, and a pairwise comparison visualized in a distance matrix (n for each group: 5). **(B)** Stacked bar charts of community composition. Operational taxonomic units (OTUs) shown were among the five most abundant OTUs in at least one sample. Time points are indicated as numbers inside the bars. *SC_TF*, sedimentation cone top fraction; *SC_BF*, sedimentation cone bottom fraction; *SC_BF_FL*, free-living population of sedimentation cone bottom fraction; *SC_BF_PA*, particle-attached population of sedimentation cone bottom fraction.

Microscopy revealed that algae and diatom shells had settled after 3 h. The supernatant still contained particles composed of extracellular polymeric substances but was dominated by single cells (96–100%). Single cells were also abundant in the bottom fraction (63.3–99%, Table 1). Bacterial communities of unfractionated seawater and of the top fraction in cones were not significantly different in OTU diversity and relative read abundance (pairwise comparison, *P* > 0.05, Figure 7, Supplementary Table 9 and Supplementary Figure 4). Only 403–487 OTUs were detected in the bottom fractions, with half of the OTU numbers detected in unfractionated seawater and the supernatant (742–1,079) (Supplementary Figure 4). The reduced richness was balanced by a larger evenness resulting in no change in the overall diversity (Supplementary Figure 4).

The settled fractions at time points 4 and 5 were rich in SAR11 clade Ia as most abundant and slightly enriched OTU compared with seawater. High enrichment factors were observed for *Psychrobacter*, *Erythrobacter*, *Rheinheimera*, *Halomonas*, *Pseudomonas*, *Oleispira*, and *Methylobacterium*, all with ≥0.1% relative read abundance in seawater (Figure 3). Among the less frequent OTUs (relative read abundance in seawater <0.1%) with enrichment factors >10 were OTUs affiliating with the genera *Roseobacter*, *Francisella*, *Acinetobacter*, and *Pirellula*.

### Second Separation of the Settled Fractions by Centrifugation

At two time points, the sedimentation cone bottom fractions collected after 3 h of settlement were resuspended in artificial seawater and separated for a second time by centrifugation. Most cells were recovered in the resulting supernatants. Overall, the number of particle-attached cells increased with the start of the phytoplankton bloom (time point 1 versus 3) (Table 1 and Supplementary Table 3). OTU communities obtained by the secondary separation were different from each other and from all other communities (Figure 7A). Three groups of taxa were identified and categorized as present in both fractions or enriched in either the supernatant or the pellet. OTUs with an enrichment and ≥0.1% relative read abundance in the supernatant included SAR11 clades Ia, Ib, I uncultured, II and III, SAR116, SUP05 group, *Oleibacter*, uncultured Nitrincolaceae, *Lentimonas*, flavobacterial marine groups NS7 and NS9, and *Puniceicoccaceae* marine group (Figure 3). OTUs specifically enriched in the pellet samples and with relative read abundances of ≥ 0.5% were *Lutimonas*, *Fusobacterium*, *Ca.* Megaira, and *Pseudophaeobacter* (Figure 3). Taxa enriched in the pellet and supernatant were in a decreasing order of relative read abundance *Pseudoalteromonas*, *Olleya*, *Nonlabens*, *Erythrobacter*, *Sulfitobacter*, *Psychrobacter*, *Flavobacterium*, *Sphingorhabdus*, *Shewanella*, *Vibrio*, *Cellulophaga*, *Pseudomonas*, *Loktanella*, *Rheinheimera*, and *Lacinutrix* (Figure 3).

## Discussion

Sequential filtration is easily performed, is widely used, and separates particle-attached, large, and aggregated bacteria well from small, single-celled, free-living microorganisms (Bidle and Fletcher, 1995; Crump et al., 1999; Ayo et al., 2001; Crespo et al., 2013; Bižić-Ionescu et al., 2014). For the enrichment of particle-associated and phycosphere bacteria, alternative separation techniques are necessary. In this study, we introduced Imhoff sedimentation cones, which enable a fractionation that targets chemotactic, free-living, motile bacteria and particle-attached microbiomes in one fraction. The taxon abundance distribution in separation experiments by filtration, centrifugation, and sedimentation cones resulted in five technically defined OTU groups: a mechanosensitive group, a more mechanically robust group of free-living bacteria, and three particle group fractions (Figure 3).

Our OTU analysis showed microbial communities in the filtration fractions similar to those observed in earlier sequential filtration experiments at Helgoland (Bižić-Ionescu et al., 2014) and other coastal seas (Mestre et al., 2017, 2020). Filtration removed particles efficiently from the seawater, together with large, filamentous and rosette-forming free-living bacteria, related to the genera *Tenacibaculum* (Avendaño-Herrera et al., 2006) and *Polaribacter* (Nedashkovskaya et al., 2013), as well as the phylum *Planctomycetes* (Ward, 2010). Also, the collection, albeit in small amounts, of small free-living non-motile bacteria, like *Ulvibacter* [now *Ca.* Prosiliicoccus vernus (Francis et al., 2019)] or *Aurantivirga* (Song et al., 2015), on particle filters has been reported before (Bižić-Ionescu et al., 2014).

We observed that the cell recovery in the 3–0.2-μm filter fraction was the lowest of all free-living fractions among the separation techniques (Table 1). Mechanical stress during sequential filtration (Ferguson et al., 1984) is likely causing the significant cell loss of a group of free-living bacteria referred to as OTU group I (Figure 3). In contrast, OTUs of this group were recovered from the supernatant of sedimentation cones (top fraction). The degree of depletion based on mechanical stress varied among different OTUs. For example, flavobacterial marine group NS4 and the gammaproteobacterial RS62 and OM43 maintained their relative read abundance, whereas the bacteria affiliated with *Aurantivirga*, *Formosa*, flavobacterial marine clade NS5, and *Hellea* seemed to be more fragile. Mechanical sensitivity in marine cells has been previously shown in *Formosa*-related strains, which have large appendages formed by biopearling on the cell surface (Fischer et al., 2019). Furthermore, atomic force microscopy suggested that approximately 30% of all seawater bacteria might be connected by cell surface extensions (Malfatti and Azam, 2009). In any case, loss of mechano-sensitive cells needs to be considered as a bias intrinsic to sequential filtration, best by comparative cell counting of fractions.

The mathematical consequence of a loss of a group of cells is the enrichment of a second, more mechano-stabile group. This OTU group II was enriched in the 3–0.2 μm fraction, but also in the 10–3 μm fraction, and the supernatant that was obtained by centrifugal separation of the sedimentation cone bottom fraction (Figure 3). The latter enrichment was surprising, since many cultured representatives of these taxa are considered non-motile species including, for example, SAR116, SAR92, and the SAR11 clades (Connon and Giovannoni, 2002; Rappé et al., 2002; Oh et al., 2010). SAR11 clades are the most abundant bacteria in the ocean. They are generally considered to be free-living, but they have been detected previously in particle fractions of sequential filtrations, especially in the cold season (Mestre et al., 2020). Their cells have less hydrophobic cell surfaces properties than other planktonic cells, a property that was linked to grazing avoidance (Dadon-Pilosof et al., 2017). It is possible that these surface properties facilitated attachment to algal particles while these sink and collide with the bacterial cells. In our experiments, cells of this group became free-living after resuspension of sedimentation cone bottom fractions, suggesting a loose nature of their particle association. Still, the adsorption to particles could provide cells like SAR11 with transient access to elevated nutrient concentrations and may contribute to the ecological success of this clade and other non-motile free-living planktonic bacteria.

The large size fractions, recovered by sequential filtration, could be further categorized according to three different distribution patterns in the cone experiments: absence in the bottom fraction of sedimentation cones (OTU group III), presence in both free-living and particle-attached subfractions of the cones’ bottom fractions (OTU group IV), and presence only in the particle-attached subfraction of the bottom fraction (OTU group V).

Absence in the Imhoff cone bottom fraction (OTU group III) was to be expected for neutral buoyant large bacteria and aggregate-forming bacteria, but also for bacteria attached to non-rapidly sinking particles. For all of these bacteria, the sinking rates were too slow to be collected at the bottom of the Imhoff cone. This OTU group III encompassed the verrucomicrobial genus *Persicirhabdus*, but also *Algibacter*, *Lewinella*, *Polaribacter*, *Leucothrix*, *Psychromonas*, *Sneathiella*, *Tenacibaculum*, and several *Planctomycetes* (Figure 3). Many of these taxa are large or filamentous cells or do form cell aggregates. Smaller cells in OTU group III (e.g., some other *Polaribacter* strains; reviewed in Nedashkovskaya et al., 2013) may be attached to non- or slowly sinking particles, for example, those consisting of transparent extracellular polymeric substances (Passow, 2002).

Large size fraction bacteria of group IV were enriched both in the supernatant and in the pellet subfraction of the Imhoff sedimentation cone bottom fractions, i.e., *Cellulophaga*, *Dokdonia*, *Sulfitobacter*, *Rheinheimera*, and *Shewanella*. Thus, OTU group IV comprised many free-living chemosensory motile taxa, predominantly alphaproteobacterial *Rhodobacteraceae* and *Gammaproteobacteria* (Seymour et al., 2017). Chemosensory motile bacteria have developed a range of fine-tuned strategies to deal with the limited life span of a particle. They often stay on or close to a particle only during nutrient-rich conditions, before they swim to the next particle (Pedrós-Alió and Brock, 1983; Ghiglione et al., 2007; Grossart, 2010; Yawata et al., 2014). Their simultaneous presence as free-living and as attached cell is the consequence of this highly dynamic response to substrate availability (Seymour et al., 2017). Microdiversity within these genera may also contribute to the presence in both fractions. The V3V4 amplicon is very variable; often over 100 OTUs (>98% sequence identity) per sample were assigned to a genus-based OTU (classified as >93% sequence identity, the recommended threshold by SILVA). To which extent this microdiversity can be used to resolve the physiology of closely related species characterized by >98% sequence identity in the V3V4 region awaits exploration.

*Flavobacteriia* and *Alphaproteobacteria* have been identified as particle-preferring bacteria, as they are often enriched in particle-attached fractions, in contrast to *Gammaproteobacteria* (Crump et al., 1999; Bižić-Ionescu et al., 2014). We found a specialization within these classes, even within families. The *Flavobacteriaceae Algibacter*, *Algitalea*, *Flavimarina*, and several *Polaribacter* were enriched in particle fractions of filtrations (group III), but not in the sedimentation cone bottom fraction, unlike flavobacterial OTUs affiliating to *Lutimonas* and *Winogradskyella* (group V). This specialization was also observed in families within alphaproteobacterial *Rhodobacteraceae*, where the pattern showed a range from free-living *Planktomarina* and *Planktotalea* over particle-associated taxa like *Loktanella*, *Roseobacter*, and *Sulfitobacter* to particle-attached cells like *Paracoccus*, *Pseudophaeobacter*, and *Roseovarius* (Figure 3). These observations highlight the individual lifestyles and niche occupations, which have already been shown within clades, e.g., SAR11 (Dadon-Pilosof et al., 2017; Mestre et al., 2020); genera, e.g., *Vibrio* (Thompson et al., 2005; Hunt et al., 2008); and even species, e.g., *Gordona hydrophobica* (Moormann et al., 1997).

Both sequential filtration and particle collection in sedimentation cones have advantages and disadvantages. The mechanical stress in sequential filtration can lead to a quantifiable cell loss, and unspecific binding of cells to particles and filters can give misleading results. These problems can be avoided by using gravitational settlement of particles.

Sedimentation cones fill an intermediate gap between filtration of a sample and oceanic instruments for particle collection, the sediment traps (McDonnell et al., 2015). The latter collect settable particles of larger volumes over longer timescales than cones and usually stop biological processes such as heterotrophic particle mineralization or grazing by adding toxins (Davis, 1967; Knauer et al., 1984). Nowadays, non-lethal traps are applied using a brine solution (Fontanez et al., 2015). Addition of a particle separator has given access to *in situ* measurements of bacterial heterotrophy (Boyd et al., 2015). The autonomous separation of water samples with sediment cones on an oceanic instrument, i.e., the design of a multiconer similar to CTD rosettes or multicorer, may in the future provide particles and their associated bacteria for determination of *in situ* activities, which is desired for a better understanding of the oceanic carbon flux.

Sedimentation cones require a particle or cell density higher than seawater for enrichment by natural gravitation. Large cells and particles with a density equal to seawater will not be collected; i.e., particles made of extracellular polymeric substances and colloidal particles, which both have a low sinking rate. Freshly formed particles, particles of spherical shape and sizes of 80 to 400 μm, and fecal pellets have sinking rates of >3, 9, and >5 m day^–1^, respectively, and can be collected in short times. However, individual diatom species have sinking rates of 0.05–10 m day^–1^ and may in some cases not be collectable (Bach et al., 2012; Turner, 2015). Hence, the usage of sedimentation cones in marine particle research requires initially optimization experiments to collect the desired particles and microscopic particle characterization. Short sedimentation times are preferred to minimize selective microbial growth and mortality by grazing or viral lysis leading to a community shift known as bottle effect. The non-lethal particle collection in addition to low settlement times in sedimentation cones allows for subsequent cultivation of the particle-associated microbiome, which remains understudied compared with the cultivation of microorganisms using seawater as source (e.g., Alejandre-Colomo et al., 2020).

In summary, gravitational sedimentation in Imhoff cones allowed chemotactic free-living bacteria, like *Shewanella* and *Pseudoalteromonas*, to follow the particles and to end up in the same fraction as bacteria attached to rapidly sinking particles. A further separation of these communities could be partially obtained by subsequent centrifugation of the resuspended bottom fractions. We conclude that the characterization of particle-associated bacteria may best be performed by multiple separation techniques, at least a combination of sequential filtration and sedimentation cones. With an appropriate consideration of the advantages and disadvantages of each technology, further studies might obtain novel insights into the ecology of planktonic microorganisms. Hypotheses, thereby generated on spatial niches of bacterial species on different classes of particles, may subsequently be tested by microscopic studies.

## Data Availability Statement

The data presented in the study is deposited in the European Nucleotide Archive (ENA, https://www.ebi.ac.uk/) repository, accession number PRJEB41742.

## Author Contributions

AH and JH planned the study, analyzed the data, and wrote the manuscript, both with contributions from GR and RA. AH performed the sampling and laboratory work. All authors approved the submitted manuscript.

## Conflict of Interest

The authors declare that the research was conducted in the absence of any commercial or financial relationships that could be construed as a potential conflict of interest.

## Footnotes

1 https://mpgc.mpipz.mpg.de/home/
