# Supplementary material for: Particle Collection in Imhoff Sedimentation Cones Enriches Both Motile Chemotactic and Particle-Attached Bacteria
Source: Front Microbiol. 2021 Apr 1;12:643730. doi: 10.3389/fmicb.2021.643730 (PMC8047139; doi:10.3389/fmicb.2021.643730)
Supplement: Supplementary file 1 [file Table_1.DOCX]

| Supplementary Table 1. Total cell counts of seawater and fractionated free-living and top fractions (FL and TF) during a phytoplankton spring bloom off Helgoland (North Sea) in 2018. | | | | | |
| --- | --- | --- | --- | --- | --- |
| Time point/  Julian day | Chl *a*  (µg L^-1^) | Seawater  (x 10^5^ cells ml^-1^) | F_0.2µm  (x 10^5^ cells ml^-1^) | C_FL  (x 10^5^ cells ml^-1^) | SC_TF  (x 10^5^ cells ml^-1^) |
| 1/ 102 | 1.2 | 6.04 ± 0.75 | 4.38 ± 0.48 | 3.83 ± 0.83 | 5.62 ± 0.11 |
| 2/ 109 | 2.0 | 7.57 ± 0.96 | 3.39 ± 0.95 | 5.89 ± 0.13 | 6.59 ± 0.28 |
| 3/ 115 | 6.1 | 11.00 ± 0.90 | 7.38 ± 0.22 | 8.08 ± 0.16 | 7.78 ± 0.14 |
| 4/ 128 | 2.4 | 14.41 ± 2.05 | 7.07 ± 0.34 | -- | 10.05 ± 0.41 |
| 5/ 142 | 6.3 | 24.72 ± 2.50 | 17.09 ± 0.67 | -- | 17.67 ± 0.64 |

C: centrifugation, F: sequential filtration (3-0.2 µm), SC: sedimentation cone.
