# Supplementary material for: Particle Collection in Imhoff Sedimentation Cones Enriches Both Motile Chemotactic and Particle-Attached Bacteria
Source: Front Microbiol. 2021 Apr 1;12:643730. doi: 10.3389/fmicb.2021.643730 (PMC8047139; doi:10.3389/fmicb.2021.643730)
Supplement: Supplementary file 2 [file Table_2.DOCX]

**Supplementary Table 2.** PERMANOVA of unfractionated seawater and sequentially filtered seawater in the range of > 10 µm, 10 – 3 µm, and 3 – 0.2 µm (n for each group: 5). Samples were obtained between March and May 2018 off Helgoland (54°11’03”N, 7°54’00”E).

|  | Df | Sums of squares | Mean squares | F Model | R^2^ | Pr(>F) | Significance |
| --- | --- | --- | --- | --- | --- | --- | --- |
| Dataset | 3 | 1.5697 | 0.52323 | 4.9133 | 0.4795 | 0.001 | 0.0001 |
| Residuals | 16 | 1.7039 | 0.10649 |  | 0.5205 |  |  |
| Total | 19 | 3.2736 |  |  | 1.00000 |  |  |

DF: degrees of freedom, n= number of samples.
