# Supplementary material for: Particle Collection in Imhoff Sedimentation Cones Enriches Both Motile Chemotactic and Particle-Attached Bacteria
Source: Front Microbiol. 2021 Apr 1;12:643730. doi: 10.3389/fmicb.2021.643730 (PMC8047139; doi:10.3389/fmicb.2021.643730)
Supplement: Supplementary file 3 [file Table_3.DOCX]

| **Supplementary Table 3.** Local cell environment on filters obtained during a phytoplankton spring bloom off Helgoland in 2018. | | | | | | | | | | | | | |
| --- | --- | --- | --- | --- | --- | --- | --- | --- | --- | --- | --- | --- | --- |
|  |  | **Cell environment on filter (%)** | | | | | | |  | **Counts (no. of)** | | |  |
|  |  | **Single** | **TEP** | **Algae** | **Diatom** | **Cluster** | **Chain** | **Aggregate** |  | **Particles** |  | **DAPI signals** |  |
| **TP1** | **C_FL** | 99.96 | 0.00 | 0.04 | 0.00 | 0.00 | 0.00 | 0.00 |  | 1 |  | 2453 |  |
|  | **C_PA** | 98.53 | 0.37 | 1.09 | 0.00 | 0.00 | 0.00 | 0.00 |  | 20 |  | 3471 |  |
|  | **F_0.2µm** | 98.32 | 1.49 | 0.13 | 0.00 | 0.00 | 0.00 | 0.07 |  | 65 |  | 6123 |  |
|  | **F_3µm** | 46.71 | 50.90 | 0.60 | 0.00 | 0.00 | 0.00 | 1.80 |  | 89 |  | 334 |  |
|  | **F_10µm** | 34.78 | 58.94 | 0.48 | 0.00 | 0.00 | 1.93 | 3.86 |  | 55 |  | 207 |  |
|  | **SC_TF** | 98.15 | 1.77 | 0.08 | 0.00 | 0.00 | 0.00 | 0.00 |  | 48 |  | 3896 |  |
|  | **SC_BF** | 99.02 | 0.77 | 0.21 | 0.00 | 0.00 | 0.00 | 0.00 |  | 25 |  | 3871 |  |
|  | **SC_BF_FL** | 92.97 | 4.96 | 1.41 | 0.00 | 0.67 | 0.00 | 0.00 |  | 22 |  | 1493 |  |
|  | **SC_BF_PA** | 90.77 | 8.62 | 0.62 | 0.00 | 0.00 | 0.00 | 0.00 |  | 18 |  | 650 |  |
| **TP2** | **C_FL** | 99.59 | 0.28 | 0.03 | 0.00 | 0.00 | 0.10 | 0.00 |  | 6 |  | 4077 |  |
|  | **C_PA** | 94.66 | 4.15 | 1.19 | 0.00 | 0.00 | 0.00 | 0.00 |  | 23 |  | 674 |  |
|  | **F_0.2µm** | 98.70 | 0.85 | 0.13 | 0.00 | 0.00 | 0.00 | 0.31 |  | 37 |  | 8682 |  |
|  | **F_3µm** | 32.14 | 56.01 | 0.00 | 0.00 | 3.64 | 8.21 | 0.00 |  | 271 |  | 2228 |  |
|  | **F_10µm** | 10.23 | 77.00 | 0.00 | 12.54 | 0.23 | 0.00 | 0.00 |  | 74 |  | 2130 |  |
|  | **SC_TF** | 99.26 | 0.47 | 0.27 | 0.00 | 0.00 | 0.00 | 0.00 |  | 17 |  | 3859 |  |
|  | **SC_BF** | 91.37 | 4.02 | 0.02 | 1.80 | 0.09 | 0.20 | 2.50 |  | 105 |  | 4601 |  |
| **TP3** | **C_FL** | 99.10 | 0.41 | 0.24 | 0.00 | 0.00 | 0.11 | 0.13 |  | 21 |  | 5330 |  |
|  | **C_PA** | 98.07 | 1.31 | 0.61 | 0.00 | 0.00 | 0.00 | 0.00 |  | 18 |  | 1142 |  |
|  | **F_0.2µm** | 100.00 | 0.00 | 0.00 | 0.00 | 0.00 | 0.00 | 0.00 |  | 0 |  | 3863 |  |
|  | **F_3µm** | 34.38 | 57.44 | 1.05 | 0.00 | 0.00 | 5.24 | 1.89 |  | 96 |  | 477 |  |
|  | **F_10µm** | 11.73 | 65.34 | 9.66 | 3.09 | 9.79 | 0.39 | 0.00 |  | 125 |  | 776 |  |
|  | **SC_TF** | 96.30 | 1.12 | 1.67 | 0.90 | 0.00 | 0.00 | 0.00 |  | 42 |  | 4541 |  |
|  | **SC_BF** | 94.37 | 5.63 | 0.00 | 0.00 | 0.00 | 0.00 | 0.00 |  | 9 |  | 284 |  |
|  | **SC_BF_FL** | 91.81 | 6.24 | 1.95 | 0.00 | 0.00 | 0.00 | 0.00 |  | 43 |  | 1490 |  |
|  | **SC_BF_PA** | 26.04 | 33.33 | 40.63 | 0.00 | 0.00 | 0.00 | 0.00 |  | 41 |  | 192 |  |
| **TP4** | **F_0.2µm** | 99.61 | 0.29 | 0.06 | 0.00 | 0.00 | 0.00 | 0.04 |  | 12 |  | 8560 |  |
|  | **F_3µm** | 70.04 | 19.49 | 5.23 | 0.00 | 1.11 | 4.12 | 0.00 |  | 117 |  | 898 |  |
|  | **F_10µm** | 47.18 | 50.91 | 0.00 | 0.00 | 1.44 | 0.48 | 0.00 |  | 93 |  | 1045 |  |
|  | **SC_TF** | 98.62 | 1.14 | 0.24 | 0.00 | 0.00 | 0.00 | 0.00 |  | 24 |  | 5364 |  |
|  | **SC_BF** | 97.33 | 2.11 | 0.56 | 0.00 | 0.00 | 0.00 | 0.00 |  | 10 |  | 711 |  |
| **TP5** | **F_0.2µm** | 98.68 | 1.03 | 0.05 | 0.00 | 0.00 | 0.00 | 0.24 |  | 23 |  | 6227 |  |
|  | **F_3µm** | 63.77 | 24.65 | 4.93 | 0.00 | 0.00 | 3.64 | 3.00 |  | 86 |  | 933 |  |
|  | **F_10µm** | 42.66 | 14.87 | 6.33 | 0.00 | 34.47 | 0.44 | 1.23 |  | 84 |  | 3174 |  |
|  | **SC_TF** | 100.00 | 0.00 | 0.00 | 0.00 | 0.00 | 0.00 | 0.00 |  | 0 |  | 3004 |  |
|  | **SC_BF** | 63.35 | 35.81 | 0.84 | 0.00 | 0.00 | 0.00 | 0.00 |  | 100 |  | 955 |  |
| BF: bottom fraction, C: centrifugation, F_0.2µm: filtered fraction 3-0.2 µm, F_3µm: filtered fraction 10-3µm, F_10µm: filtered fraction > 10 µm, FL: free-living fraction, No. particles: Number of particles, PA: particle-attached fraction, SC: sedimentation cone, TF: top fraction, TP: sample time point (Julian Days 102, 109, 115, 128, and 142). | | | | | | | | | | | | | |
