# Supplementary material for: Particle Collection in Imhoff Sedimentation Cones Enriches Both Motile Chemotactic and Particle-Attached Bacteria
Source: Front Microbiol. 2021 Apr 1;12:643730. doi: 10.3389/fmicb.2021.643730 (PMC8047139; doi:10.3389/fmicb.2021.643730)
Supplement: Supplementary file 4 [file Table_4.DOCX]

**Supplementary Table 4.** Bacterial community composition. Relative read abundances of all OTUs in all fractions of all 2018 samples (Excel sheet, electronic supplementary material 1). Abbreviations of the file names: 20µm: plankton net fraction 20 µm, 80µm: plankton net fraction 80 µm, BF: bottom fraction, C: centrifugation, F_0.2µm: filtered fraction 3-0.2 µm, F_3µm: filtered fraction 10-3 µm, F_10µm: filtered fraction > 10 µm, FL: free-living fraction, PA: particle-attached fraction, SC: sedimentation cone, TF: top fraction. Numbers behind the sample names indicate the sampling time points (Julian Days 102, 109, 115, 128, and 142).
