# Supplementary material for: Particle Collection in Imhoff Sedimentation Cones Enriches Both Motile Chemotactic and Particle-Attached Bacteria
Source: Front Microbiol. 2021 Apr 1;12:643730. doi: 10.3389/fmicb.2021.643730 (PMC8047139; doi:10.3389/fmicb.2021.643730)
Supplement: Supplementary file 6 [file Table_6.DOCX]

**Supplementary Table 6.** PERMANOVA of three sample groups from 2017: (i) 24 h sedimentation cone bottom fraction > 3µm (n= 8); (ii) directly filtered > 3µm (n= 4); (iii) directly filtered < 3 µm and > 0.2 µm (n= 4), obtained at the end of March (Julian day 88) and beginning of April 2017 (Julian day 94) off Helgoland (54°11’03”N, 7°54’00”E).

|  | Df | Sums of squares | Mean squares | F Model | R^2^ | Pr(>F) | Significance |
| --- | --- | --- | --- | --- | --- | --- | --- |
| Dataset | 2 | 2.4469 | 1.22346 | 10.077 | 0.001 | 0.60788 | 0.0001 |
| Residuals | 13 | 1.5784 | 0.12141 |  | 0.39212 |  |  |
| Total | 15 | 4.0253 |  |  | 1.00000 |  |  |

DF: degrees of freedom, n= number of samples.
