# Supplementary material for: Particle Collection in Imhoff Sedimentation Cones Enriches Both Motile Chemotactic and Particle-Attached Bacteria
Source: Front Microbiol. 2021 Apr 1;12:643730. doi: 10.3389/fmicb.2021.643730 (PMC8047139; doi:10.3389/fmicb.2021.643730)
Supplement: Supplementary file 7 [file Table_7.DOCX]

**Supplementary Table 7.** Pairwise comparisons using permutation MANOVAs on a distance matrix of three sample groups, (i) 24h sedimentation cone bottom fraction > 3 µm (SC_BF_3µm, n= 8); (ii) directly filtered > 3 µm (F_>3µm, n= 4); (iii) directly filtered < 3 µm and > 0.2 µm (F_0.2µm, n= 4), obtained at the end of March and beginning of April 2017 off Helgoland (54°11’03”N, 7°54’00”E).

|  | SC_BF_3µm | F_>3µm |
| --- | --- | --- |
| F_>3µm | 0.003 |  |
| F_0.2µm | 0.006 | 0.029 |
