# Supplementary material for: Particle Collection in Imhoff Sedimentation Cones Enriches Both Motile Chemotactic and Particle-Attached Bacteria
Source: Front Microbiol. 2021 Apr 1;12:643730. doi: 10.3389/fmicb.2021.643730 (PMC8047139; doi:10.3389/fmicb.2021.643730)
Supplement: Supplementary file 8 [file Table_8.DOCX]

**Supplementary Table 8.** Table of operational taxonomic units (OTUs) that reached 1% relative read abundance (shown in %) at least once in a sample. Samples were taken off Helgoland (54°11’03”N, 7°54’00”E) in 2017 during pre-bloom condition at the end of March (Julian day 88, TP1) and beginning of April (Julian day 94, TP2), as well as during a phytoplankton bloom mid-May (Julian day 135, TP3).

|  |  | **Direct filtration** | | | | | | | | | | | |  | **Sedimentation cone 24h** | | | | | | | |
| --- | --- | --- | --- | --- | --- | --- | --- | --- | --- | --- | --- | --- | --- | --- | --- | --- | --- | --- | --- | --- | --- | --- |
|  |  | **> 3µm** | | | | | | **< 3µm and > 0.2µm** | | | | | |  | **Sedimentation cone bottom fraction > 3µm** | | | | | | | |
| **Phylum** | **OTUs** | **TPa1** | **TPb1** | **TPa2** | **TPb2** | **TPa3** | **TPb3** | **TPa1** | **TPb1** | **TPa2** | **TPb2** | **TPa3** | **TPb3** |  | **TPa1** | **TPb1** | **TPc1** | **TPd1** | **TPa2** | **TPb2** | **TPc2** | **TPd2** |
| *Actino* | *Ca.* Actinomarina | 4.17 | 1.76 | 2.01 | 2.47 | 0.17 | 0.12 | 2.65 | 5.22 | 5.76 | 3.47 | 4.54 | 5.00 |  | 0.00 | 0.08 | 0.03 | 0.01 | 0.01 | 0.07 | 0.00 | 0.01 |
|  | *Ilumatobacter* | 0.11 | 1.23 | 0.10 | 0.09 | 0.02 | 0.08 | 0.05 | 0.35 | 0.12 | 0.03 | 0.03 | 0.05 |  | 0.00 | 0.03 | 0.14 | 0.04 | 0.05 | 0.02 | 0.02 | 0.06 |
|  | Sva0996 marine group | 0.32 | 2.06 | 0.91 | 0.96 | 0.25 | 0.41 | 0.62 | 2.55 | 1.10 | 0.39 | 0.42 | 0.24 |  | 0.01 | 0.02 | 0.02 | 0.03 | 0.03 | 0.06 | 0.00 | 0.01 |
| *Bacteroidetes* | Lewinella | 0.02 | 0.10 | 0.01 | 0.03 | 1.20 | 0.83 | 0.01 | 0.02 | 0.00 | 0.01 | 0.03 | 0.01 |  | 0.08 | 0.25 | 0.88 | 0.61 | 0.18 | 0.06 | 0.08 | 0.07 |
|  | *Saprospiraceae* uncultured | 0.12 | 0.28 | 0.31 | 0.16 | 3.08 | 2.10 | 0.04 | 0.04 | 0.03 | 0.05 | 0.86 | 0.74 |  | 0.03 | 0.08 | 0.48 | 0.23 | 0.11 | 0.07 | 0.67 | 0.14 |
|  | *Brumimicrobium* | 0.24 | 0.00 | 0.07 | 0.30 | 0.21 | 0.09 | 0.08 | 0.05 | 0.09 | 0.13 | 0.04 | 0.00 |  | 0.98 | 2.25 | 1.02 | 0.50 | 0.00 | 0.12 | 0.02 | 0.00 |
|  | *Fluviicola* | 4.12 | 2.55 | 11.6 | 7.06 | 4.38 | 1.33 | 0.89 | 1.19 | 2.22 | 1.39 | 2.24 | 0.28 |  | 0.06 | 0.28 | 0.24 | 0.06 | 1.46 | 3.04 | 0.33 | 1.14 |
|  | *Luteibaculum* | 0.01 | 0.00 | 0.04 | 0.00 | 0.01 | 0.01 | 0.00 | 0.01 | 0.01 | 0.00 | 0.01 | 0.00 |  | 0.00 | 0.12 | 2.54 | 0.33 | 0.00 | 0.00 | 0.00 | 0.00 |
|  | *Cryomorphaceae* uncultured | 3.23 | 1.67 | 1.69 | 1.78 | 1.00 | 0.54 | 2.68 | 4.91 | 3.80 | 1.89 | 3.13 | 3.79 |  | 0.03 | 0.18 | 0.28 | 0.08 | 0.03 | 0.14 | 0.04 | 0.03 |
|  | *Aquibacter* | 0.81 | 0.65 | 0.85 | 0.42 | 2.69 | 2.25 | 0.26 | 0.44 | 0.33 | 0.19 | 0.60 | 0.15 |  | 0.03 | 0.16 | 0.14 | 0.06 | 0.11 | 0.28 | 0.14 | 0.19 |
|  | *Aurantivirga* | 0.53 | 0.00 | 0.07 | 0.27 | 0.51 | 0.24 | 0.35 | 0.67 | 0.26 | 0.21 | 2.89 | 0.74 |  | 0.00 | 0.09 | 0.03 | 0.01 | 0.00 | 0.05 | 0.00 | 0.02 |
|  | *Bizionia* | 0.09 | 0.04 | 0.16 | 0.04 | 0.13 | 0.12 | 0.03 | 0.01 | 0.01 | 0.01 | 0.02 | 0.00 |  | 0.27 | 0.72 | 2.59 | 0.61 | 0.17 | 0.62 | 0.40 | 0.37 |
|  | *Dokdonia* | 0.19 | 0.00 | 0.02 | 0.07 | 1.97 | 0.53 | 0.04 | 0.04 | 0.02 | 0.05 | 0.14 | 0.07 |  | 0.21 | 0.32 | 0.60 | 0.16 | 0.14 | 0.11 | 0.17 | 0.29 |
|  | *Flavobacterium* | 0.07 | 0.00 | 0.00 | 0.14 | 0.71 | 0.20 | 0.05 | 0.02 | 0.00 | 0.07 | 0.08 | 0.02 |  | 0.02 | 0.23 | 0.20 | 0.13 | 2.84 | 6.44 | 0.75 | 0.33 |
|  | *Gramella* | 0.18 | 0.29 | 0.14 | 0.03 | 0.02 | 0.02 | 0.04 | 0.01 | 0.02 | 0.02 | 0.01 | 0.01 |  | 2.04 | 2.01 | 5.89 | 4.24 | 0.89 | 0.60 | 1.57 | 0.29 |
|  | *Lacinutrix* | 0.03 | 0.00 | 0.00 | 0.01 | 0.25 | 0.31 | 0.01 | 0.01 | 0.00 | 0.01 | 0.04 | 0.02 |  | 0.19 | 0.12 | 0.35 | 0.14 | 1.64 | 0.08 | 0.12 | 0.09 |
|  | *Maribacter* | 0.21 | 0.00 | 0.00 | 0.13 | 0.20 | 0.12 | 0.08 | 0.06 | 0.01 | 0.08 | 0.06 | 0.02 |  | 0.05 | 0.09 | 0.46 | 0.31 | 0.45 | 0.12 | 1.26 | 0.22 |
|  | *Mesonia* | 0.06 | 0.00 | 0.00 | 0.01 | 0.00 | 0.00 | 0.01 | 0.00 | 0.01 | 0.01 | 0.00 | 0.00 |  | 0.28 | 0.34 | 1.20 | 0.32 | 0.00 | 0.02 | 0.03 | 0.02 |
|  | *Nonlabens* | 0.11 | 0.00 | 0.03 | 0.10 | 0.05 | 0.02 | 0.02 | 0.01 | 0.00 | 0.03 | 0.01 | 0.00 |  | 0.31 | 1.00 | 0.93 | 0.28 | 0.25 | 0.13 | 0.10 | 0.01 |
|  | NS2b marine group | 1.33 | 0.23 | 0.61 | 1.01 | 0.20 | 0.06 | 1.11 | 1.05 | 1.10 | 0.91 | 0.95 | 1.03 |  | 0.00 | 0.03 | 0.01 | 0.00 | 0.03 | 0.05 | 0.00 | 0.00 |
|  | NS3a marine group | 2.09 | 0.92 | 0.03 | 1.39 | 0.19 | 0.03 | 1.91 | 1.97 | 0.55 | 1.49 | 0.80 | 0.24 |  | 0.00 | 0.07 | 0.00 | 0.01 | 0.04 | 0.09 | 0.00 | 0.00 |
|  | NS4 marine group | 1.81 | 2.91 | 3.87 | 3.84 | 0.43 | 0.28 | 4.95 | 7.90 | 8.28 | 4.87 | 2.66 | 2.52 |  | 0.01 | 0.15 | 0.02 | 0.01 | 0.16 | 0.16 | 0.01 | 0.01 |
|  | NS5 marine group | 3.01 | 4.94 | 3.69 | 2.67 | 0.71 | 0.30 | 3.52 | 7.36 | 7.14 | 3.05 | 3.71 | 4.81 |  | 0.01 | 0.13 | 0.02 | 0.01 | 0.02 | 0.16 | 0.02 | 0.01 |
|  | *Olleya* | 0.05 | 0.00 | 0.01 | 0.03 | 0.38 | 0.36 | 0.02 | 0.02 | 0.01 | 0.01 | 0.05 | 0.01 |  | 0.18 | 0.28 | 0.90 | 0.16 | 3.25 | 0.93 | 1.80 | 1.07 |
|  | *Polaribacter 4* | 0.09 | 0.00 | 0.00 | 0.07 | 0.17 | 0.15 | 0.05 | 0.02 | 0.01 | 0.04 | 0.05 | 0.03 |  | 1.01 | 1.84 | 0.95 | 0.32 | 0.30 | 0.13 | 0.10 | 0.25 |
|  | *Pseudofulvibacter* | 0.04 | 0.31 | 0.00 | 0.07 | 2.89 | 2.34 | 0.02 | 0.01 | 0.01 | 0.02 | 0.17 | 0.05 |  | 0.01 | 0.02 | 0.06 | 0.01 | 0.00 | 0.03 | 0.04 | 0.01 |
|  | *Tenacibaculum* | 0.30 | 0.05 | 0.30 | 0.15 | 1.51 | 1.15 | 0.12 | 0.09 | 0.34 | 0.08 | 0.68 | 0.15 |  | 0.27 | 1.11 | 0.11 | 0.13 | 0.11 | 0.08 | 0.05 | 0.00 |
|  | *Ulvibacter* | 0.30 | 0.42 | 0.16 | 0.18 | 2.54 | 1.43 | 0.18 | 0.28 | 0.15 | 0.12 | 5.14 | 6.14 |  | 0.07 | 0.16 | 0.28 | 0.25 | 0.19 | 0.10 | 0.02 | 0.08 |
|  | *Winogradskyella* | 0.15 | 0.03 | 0.11 | 0.08 | 0.66 | 1.77 | 0.04 | 0.06 | 0.02 | 0.03 | 0.15 | 0.11 |  | 0.13 | 0.24 | 0.79 | 0.24 | 0.05 | 0.09 | 0.03 | 0.11 |
|  | NS9 marine group | 1.30 | 0.30 | 0.17 | 0.66 | 1.71 | 1.59 | 0.75 | 0.55 | 0.52 | 0.60 | 1.58 | 0.54 |  | 0.04 | 0.15 | 0.07 | 0.02 | 0.02 | 0.08 | 0.02 | 0.02 |
| *Cyan* | Synechococcus CC9902 | 0.31 | 1.05 | 0.57 | 0.31 | 0.68 | 0.49 | 0.15 | 1.40 | 0.46 | 0.11 | 0.74 | 0.23 |  | 0.00 | 0.02 | 0.01 | 0.00 | 0.04 | 0.02 | 0.01 | 0.01 |
| *Epsi* | *Arcobacter* | 0.14 | 0.00 | 0.00 | 0.07 | 0.44 | 0.31 | 0.06 | 0.09 | 0.02 | 0.06 | 0.25 | 0.25 |  | 1.03 | 1.12 | 0.66 | 0.26 | 0.12 | 0.17 | 0.08 | 0.16 |
| *Firm* | *Fusibacter* | 0.03 | 0.00 | 0.24 | 0.03 | 0.33 | 0.33 | 0.02 | 0.01 | 0.01 | 0.03 | 0.09 | 0.07 |  | 0.00 | 0.03 | 0.01 | 0.05 | 0.00 | 1.37 | 0.03 | 0.00 |
| *Fuso* | *Psychrilyobacter* | 0.05 | 0.00 | 0.43 | 0.05 | 0.05 | 0.09 | 0.01 | 0.00 | 0.00 | 0.02 | 0.05 | 0.14 |  | 0.07 | 1.45 | 0.71 | 8.76 | 0.01 | 0.02 | 0.04 | 0.10 |
| *Mari* | SAR406 clade | 0.15 | 0.00 | 0.85 | 0.14 | 0.02 | 0.02 | 0.31 | 0.36 | 0.67 | 0.46 | 0.22 | 1.23 |  | 0.00 | 0.01 | 0.00 | 0.00 | 0.00 | 0.01 | 0.00 | 0.00 |
| *Pate* | Gracilibacteria JGI 0000069 P22 | 0.06 | 0.01 | 0.17 | 0.03 | 0.51 | 0.26 | 0.02 | 0.15 | 0.02 | 0.02 | 0.06 | 0.25 |  | 26.9 | 2.89 | 3.70 | 10.8 | 1.61 | 0.02 | 0.26 | 0.30 |
| *Alphaproteobacteria* | *Caedibacteraceae* uncultured | 0.00 | 0.01 | 0.02 | 0.00 | 0.12 | 6.72 | 0.00 | 0.01 | 0.02 | 0.00 | 0.00 | 0.01 |  | 0.02 | 0.00 | 0.09 | 0.01 | 0.07 | 0.00 | 17.2 | 0.01 |
|  | *Hellea* | 0.83 | 0.79 | 0.14 | 0.69 | 0.79 | 0.64 | 0.97 | 1.39 | 0.99 | 0.85 | 0.48 | 0.30 |  | 0.01 | 0.05 | 0.01 | 0.00 | 0.04 | 0.04 | 0.11 | 0.03 |
|  | *Litorimonas* | 0.05 | 0.00 | 0.01 | 0.03 | 0.12 | 0.16 | 0.01 | 0.02 | 0.00 | 0.01 | 0.01 | 0.02 |  | 0.09 | 0.07 | 0.33 | 0.03 | 0.00 | 0.02 | 1.71 | 0.00 |
|  | *Kordiimonadales* uncultured | 0.14 | 1.03 | 0.00 | 0.11 | 0.05 | 0.05 | 0.14 | 0.20 | 0.13 | 0.17 | 0.29 | 0.54 |  | 0.00 | 0.00 | 0.00 | 0.00 | 0.00 | 0.01 | 0.00 | 0.00 |
|  | SAR116 clade | 1.99 | 1.97 | 2.58 | 2.06 | 0.17 | 0.19 | 2.88 | 2.52 | 3.40 | 3.21 | 1.37 | 2.02 |  | 0.01 | 0.08 | 0.01 | 0.00 | 0.01 | 0.09 | 0.01 | 0.08 |
|  | *Methylobacterium* | 0.00 | 1.89 | 0.77 | 0.00 | 0.00 | 0.09 | 0.00 | 0.04 | 0.05 | 0.00 | 0.00 | 0.04 |  | 0.01 | 0.00 | 0.06 | 0.02 | 0.31 | 0.00 | 0.07 | 1.05 |
|  | *Amylibacter* | 1.45 | 2.21 | 0.86 | 1.44 | 1.25 | 1.10 | 1.41 | 1.33 | 1.91 | 1.95 | 5.29 | 6.93 |  | 0.02 | 0.11 | 0.04 | 0.01 | 0.04 | 0.14 | 0.01 | 0.09 |
|  | *Ascidiaceihabitans* | 0.33 | 1.36 | 0.61 | 0.33 | 0.19 | 0.12 | 0.37 | 0.54 | 0.43 | 0.24 | 0.45 | 0.36 |  | 0.00 | 0.01 | 0.00 | 0.00 | 0.05 | 0.03 | 0.00 | 0.00 |
|  | *Lentibacter* | 0.45 | 0.17 | 0.01 | 0.49 | 0.24 | 0.38 | 0.67 | 0.41 | 0.35 | 0.75 | 1.56 | 0.46 |  | 0.00 | 0.01 | 0.01 | 0.01 | 0.00 | 0.03 | 0.05 | 0.01 |
|  | *Loktanella* | 0.08 | 0.20 | 0.04 | 0.49 | 0.23 | 0.32 | 0.03 | 0.03 | 0.01 | 0.07 | 0.04 | 0.03 |  | 0.16 | 0.13 | 1.32 | 0.40 | 4.47 | 2.25 | 3.24 | 3.28 |
|  | *Planktomarina* | 0.85 | 6.09 | 2.90 | 4.14 | 1.33 | 1.09 | 3.50 | 7.30 | 4.13 | 3.08 | 5.66 | 4.25 |  | 0.02 | 0.13 | 0.03 | 0.03 | 0.17 | 0.21 | 0.04 | 0.09 |
|  | *Roseovarius* | 0.02 | 0.01 | 0.01 | 0.03 | 0.13 | 0.23 | 0.01 | 0.01 | 0.01 | 0.01 | 0.01 | 0.01 |  | 0.52 | 0.12 | 3.68 | 0.83 | 3.69 | 0.45 | 1.01 | 1.06 |
|  | *Sulfitobacter* | 0.73 | 1.43 | 2.52 | 0.63 | 1.62 | 1.99 | 0.73 | 1.42 | 1.02 | 0.45 | 1.65 | 3.92 |  | 0.74 | 0.31 | 4.13 | 1.31 | 9.39 | 2.33 | 3.11 | 3.08 |
|  | *Rhodobacteraceae* uncultured | 0.75 | 1.48 | 0.85 | 1.43 | 1.24 | 1.62 | 1.53 | 1.44 | 1.51 | 1.60 | 1.03 | 0.67 |  | 0.15 | 0.12 | 0.79 | 0.23 | 0.72 | 0.24 | 0.44 | 0.34 |
|  | *Rhodospirillales* uncultured | 0.01 | 0.00 | 0.00 | 0.01 | 0.00 | 0.01 | 0.01 | 0.01 | 0.00 | 0.01 | 0.00 | 0.00 |  | 3.84 | 0.12 | 0.12 | 0.21 | 0.00 | 0.01 | 0.06 | 0.00 |
|  | *Kiloniella* | 0.14 | 0.56 | 0.18 | 0.12 | 3.22 | 5.16 | 0.02 | 0.02 | 0.05 | 0.04 | 0.30 | 0.10 |  | 0.00 | 0.02 | 0.02 | 0.00 | 0.01 | 0.04 | 0.01 | 0.08 |
|  | SAR11 Clade Ia | 16.2 | 16.0 | 14.3 | 19.1 | 0.68 | 0.48 | 26.9 | 17.2 | 24.3 | 26.7 | 17.4 | 25.7 |  | 0.05 | 0.27 | 0.06 | 0.05 | 0.79 | 0.48 | 0.08 | 0.06 |
|  | SAR11 Clade Ib | 2.17 | 0.13 | 5.01 | 2.82 | 0.22 | 0.05 | 2.30 | 1.46 | 3.76 | 2.37 | 1.44 | 2.99 |  | 0.00 | 0.07 | 0.00 | 0.00 | 0.01 | 0.09 | 0.01 | 0.02 |
|  | SAR11 Clade II | 2.45 | 1.97 | 2.36 | 2.33 | 0.11 | 0.05 | 2.98 | 1.58 | 2.65 | 2.90 | 0.99 | 0.93 |  | 0.00 | 0.07 | 0.00 | 0.01 | 0.06 | 0.09 | 0.00 | 0.00 |
|  | SAR11 Clade I uncultured | 2.23 | 0.14 | 0.18 | 1.10 | 0.07 | 0.08 | 2.13 | 0.70 | 0.79 | 2.41 | 1.30 | 0.53 |  | 0.00 | 0.16 | 0.00 | 0.00 | 0.02 | 0.04 | 0.00 | 0.00 |
|  | *Erythrobacter* | 0.01 | 0.01 | 0.01 | 0.02 | 0.02 | 0.02 | 0.01 | 0.02 | 0.00 | 0.01 | 0.00 | 0.00 |  | 0.12 | 0.06 | 0.86 | 0.20 | 8.83 | 0.41 | 0.47 | 2.04 |
|  | *Sphingopyxis* | 0.02 | 0.00 | 0.18 | 0.06 | 0.00 | 0.01 | 0.01 | 0.02 | 0.03 | 0.01 | 0.00 | 0.00 |  | 0.02 | 0.01 | 0.08 | 0.01 | 2.18 | 0.38 | 0.21 | 0.17 |
|  | *Sphingorhabdus* | 0.04 | 0.05 | 0.30 | 0.03 | 0.05 | 0.04 | 0.00 | 0.03 | 0.01 | 0.01 | 0.01 | 0.01 |  | 0.03 | 0.09 | 0.34 | 0.14 | 2.40 | 0.10 | 0.16 | 0.37 |
| *Gammaproteobacteria* | *Rheinheimera* | 0.03 | 0.14 | 0.00 | 0.02 | 2.72 | 0.91 | 0.02 | 0.00 | 0.00 | 0.02 | 0.12 | 0.03 |  | 0.09 | 0.27 | 0.23 | 0.11 | 0.49 | 0.14 | 0.46 | 0.43 |
|  | *Colwellia* | 2.07 | 1.15 | 1.00 | 3.56 | 14.9 | 21.5 | 0.46 | 0.16 | 0.13 | 0.32 | 0.83 | 0.38 |  | 18.2 | 16.3 | 6.89 | 5.52 | 1.21 | 3.03 | 8.03 | 4.11 |
|  | *Moritella* | 0.04 | 0.02 | 0.00 | 0.07 | 0.24 | 0.33 | 0.02 | 0.01 | 0.02 | 0.02 | 0.15 | 0.07 |  | 1.41 | 1.21 | 0.76 | 0.50 | 0.00 | 0.02 | 0.27 | 0.02 |
|  | *Pseudoalteromonas* | 1.35 | 0.44 | 0.07 | 1.30 | 0.85 | 1.10 | 0.83 | 0.24 | 0.09 | 0.85 | 0.32 | 0.12 |  | 18.9 | 20.3 | 5.23 | 21.7 | 1.20 | 2.38 | 3.84 | 1.88 |
|  | *Psychrosphaera* | 0.04 | 0.00 | 0.01 | 0.02 | 0.02 | 0.01 | 0.03 | 0.01 | 0.00 | 0.02 | 0.02 | 0.01 |  | 4.08 | 3.55 | 0.42 | 1.87 | 0.08 | 0.08 | 0.04 | 0.11 |
|  | *Shewanella* | 0.09 | 2.52 | 1.07 | 0.06 | 0.70 | 0.96 | 0.04 | 0.10 | 0.14 | 0.06 | 0.07 | 0.05 |  | 0.15 | 0.29 | 0.76 | 1.11 | 2.74 | 5.44 | 5.20 | 13.9 |
|  | OM43 clade | 2.12 | 0.60 | 2.10 | 1.40 | 0.16 | 0.18 | 2.91 | 2.53 | 2.64 | 2.98 | 0.95 | 1.11 |  | 0.01 | 0.05 | 0.01 | 0.00 | 0.12 | 0.09 | 0.02 | 0.01 |
|  | *Incertae Sedis* Family Marinicella | 0.83 | 1.50 | 1.99 | 0.06 | 0.33 | 0.20 | 0.04 | 0.13 | 0.06 | 0.01 | 0.01 | 0.02 |  | 0.11 | 0.40 | 0.88 | 0.28 | 0.51 | 0.06 | 0.03 | 0.32 |
|  | *Alcanivorax* | 0.34 | 0.01 | 0.02 | 0.10 | 0.14 | 0.19 | 0.12 | 0.03 | 0.02 | 0.09 | 0.05 | 0.03 |  | 0.06 | 0.41 | 1.28 | 0.16 | 0.04 | 0.08 | 0.07 | 0.03 |
|  | *Halomonas* | 0.00 | 4.72 | 1.62 | 0.07 | 0.01 | 0.28 | 0.00 | 0.12 | 0.16 | 0.04 | 0.01 | 0.03 |  | 0.03 | 0.12 | 0.28 | 0.18 | 0.68 | 0.28 | 0.20 | 0.59 |
|  | *Pseudohongiella* | 1.17 | 1.00 | 0.54 | 0.91 | 0.14 | 0.11 | 1.04 | 0.45 | 0.54 | 1.18 | 0.61 | 0.38 |  | 0.01 | 0.08 | 0.01 | 0.01 | 0.00 | 0.09 | 0.01 | 0.03 |
|  | *Oleispira* | 0.17 | 0.07 | 0.01 | 0.06 | 1.71 | 2.43 | 0.06 | 0.01 | 0.01 | 0.03 | 0.23 | 0.18 |  | 2.61 | 2.82 | 0.83 | 1.17 | 0.06 | 0.10 | 0.14 | 0.11 |
|  | OM182 clade | 0.97 | 0.32 | 0.47 | 0.63 | 0.10 | 0.08 | 1.18 | 0.75 | 0.58 | 1.26 | 0.66 | 0.14 |  | 0.00 | 0.03 | 0.00 | 0.00 | 0.00 | 0.05 | 0.01 | 0.00 |
|  | *Psychrobacter* | 0.76 | 0.35 | 0.27 | 1.99 | 0.17 | 0.03 | 0.20 | 0.04 | 0.04 | 0.42 | 0.14 | 0.01 |  | 1.62 | 5.68 | 3.66 | 1.50 | 7.33 | 26.6 | 3.35 | 3.89 |
|  | *Pseudomonas* | 0.12 | 0.14 | 0.10 | 0.15 | 0.11 | 0.11 | 0.09 | 0.04 | 0.05 | 0.12 | 0.07 | 0.04 |  | 0.08 | 0.14 | 0.19 | 0.08 | 24.8 | 18.2 | 5.75 | 37.6 |
|  | SAR86 clade | 4.39 | 1.45 | 1.50 | 4.06 | 0.69 | 0.32 | 6.03 | 2.43 | 2.76 | 7.67 | 6.96 | 5.64 |  | 0.02 | 0.24 | 0.02 | 0.01 | 0.15 | 0.30 | 0.02 | 0.04 |
|  | *Woeseia* | 0.41 | 1.45 | 0.89 | 0.36 | 0.09 | 0.10 | 0.03 | 0.03 | 0.02 | 0.04 | 0.02 | 0.01 |  | 0.01 | 0.02 | 0.01 | 0.01 | 0.19 | 0.08 | 0.01 | 0.07 |
|  | *Thioglobaceae* SUP05 cluster | 0.54 | 0.93 | 1.48 | 0.68 | 0.13 | 0.12 | 0.83 | 0.64 | 1.02 | 1.03 | 1.57 | 2.61 |  | 0.00 | 0.04 | 0.00 | 0.00 | 0.01 | 0.04 | 0.01 | 0.01 |
|  | *Leucothrix* | 0.05 | 0.17 | 0.11 | 0.05 | 1.90 | 1.25 | 0.02 | 0.01 | 0.01 | 0.02 | 0.07 | 0.03 |  | 0.01 | 0.03 | 0.04 | 0.01 | 0.00 | 0.03 | 1.74 | 0.03 |
|  | *Aliivibrio* | 0.05 | 0.00 | 0.01 | 0.07 | 0.15 | 0.16 | 0.04 | 0.02 | 0.01 | 0.03 | 0.03 | 0.02 |  | 0.44 | 1.74 | 0.49 | 1.79 | 0.00 | 0.57 | 0.36 | 0.07 |
|  | *Vibrio* | 0.57 | 0.19 | 0.41 | 0.64 | 1.81 | 3.18 | 0.37 | 0.13 | 0.12 | 0.39 | 0.39 | 0.22 |  | 6.29 | 14.9 | 23.0 | 24.1 | 0.47 | 10.8 | 27.1 | 9.81 |
| *Verru* | *Persicirhabdus* | 1.24 | 0.09 | 0.12 | 0.80 | 1.60 | 0.20 | 0.06 | 0.05 | 0.02 | 0.06 | 0.12 | 0.02 |  | 0.00 | 0.02 | 0.01 | 0.00 | 0.04 | 0.08 | 0.01 | 0.01 |
|  | No Relative | 2.48 | 1.11 | 3.58 | 2.10 | 2.71 | 1.56 | 5.60 | 2.90 | 4.10 | 4.73 | 2.09 | 2.14 |  | 0.71 | 0.62 | 0.24 | 0.16 | 0.99 | 0.59 | 0.24 | 1.44 |
|  | SUM | 76.7 | 77.6 | 80.4 | 81.4 | 73.5 | 76.1 | 87.7 | 85.5 | 91.2 | 88.1 | 87.3 | 92.0 |  | 94.9 | 89.3 | 83.6 | 92.9 | 88.7 | 92.2 | 93.2 | 91.8 |

*Actino: Actinobacteria, Cyan: Cyanobacteria, Epsi: Epsilonbacteraeota, Firm: Firmicutes, Fuso: Fusobacteria, Mari: Marinimicrobia, Pate: Patescibacteria, Verru: Verrucomicrobia*
