# Supplementary material for: Particle Collection in Imhoff Sedimentation Cones Enriches Both Motile Chemotactic and Particle-Attached Bacteria
Source: Front Microbiol. 2021 Apr 1;12:643730. doi: 10.3389/fmicb.2021.643730 (PMC8047139; doi:10.3389/fmicb.2021.643730)
Supplement: Supplementary file 9 [file Table_9.DOCX]

**Supplementary Table 9.** PERMANOVA of unfractionated seawater, sequentially filtered seawater in the range of > 10 µm, 10 – 3 µm, and 3 – 0.2 µm, and free-living sedimentation cone fraction (n for each group: 5). Samples were obtained between March and May 2018 off Helgoland (54°11’03”N, 7°54’00”E).

|  | Df | Sums of squares | Mean squares | F Model | R^2^ | Pr(>F) | Significance |
| --- | --- | --- | --- | --- | --- | --- | --- |
| Dataset | 4 | 1.8092 | 0.45230 | 4.0843 | 0.4496 | 0.001 | 0.0001 |
| Residuals | 20 | 2.2148 | 0.11074 |  | 0.5504 |  |  |
| Total | 24 | 4.0240 |  |  | 1.00000 |  |  |

DF: degrees of freedom, n= number of samples.
