# Supplementary material for: Particle Collection in Imhoff Sedimentation Cones Enriches Both Motile Chemotactic and Particle-Attached Bacteria
Source: Front Microbiol. 2021 Apr 1;12:643730. doi: 10.3389/fmicb.2021.643730 (PMC8047139; doi:10.3389/fmicb.2021.643730)
Supplement: Supplementary file 11 [file Data_Sheet_2.PDF]

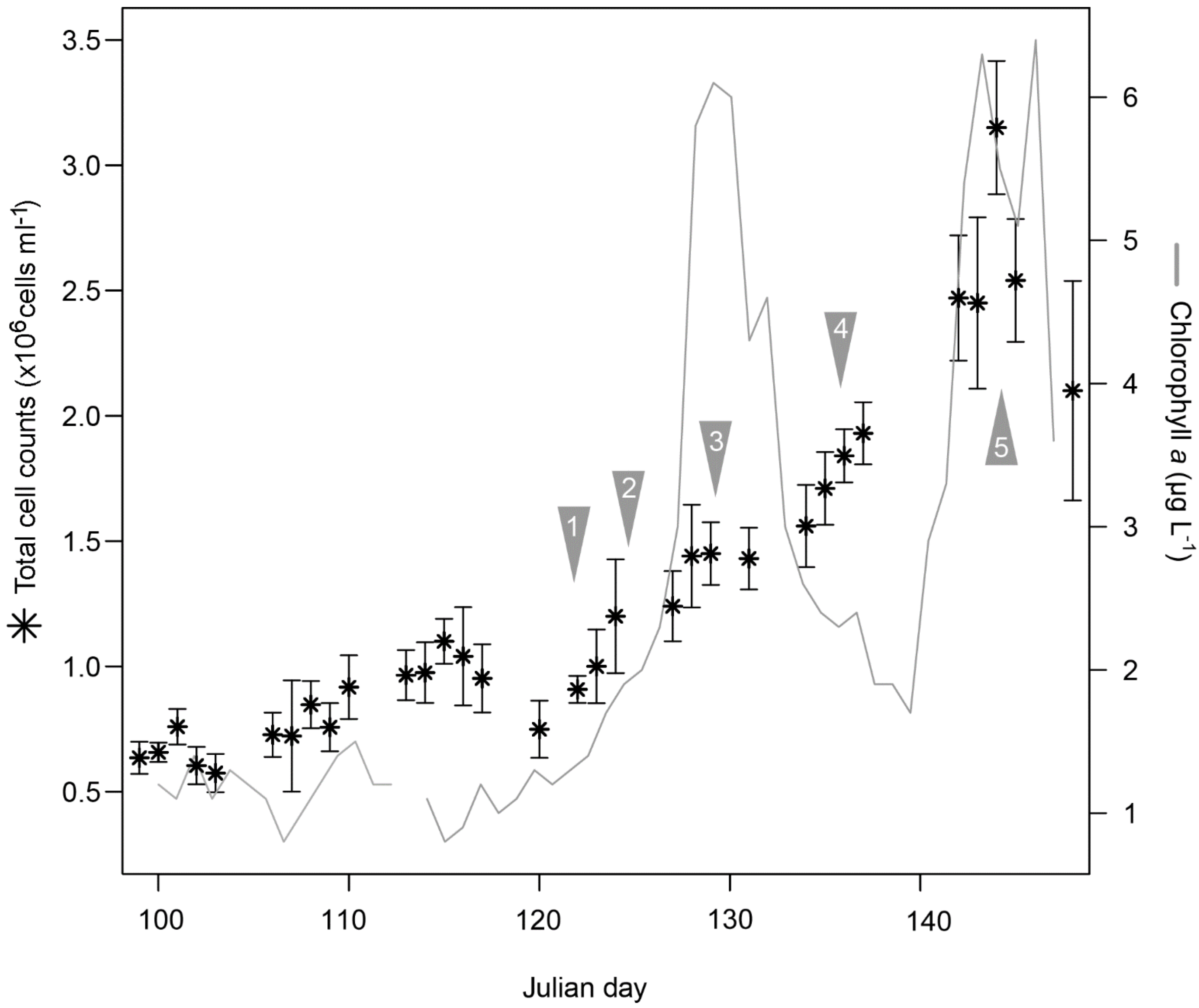

Supplementary Figure 1. Development of a phytoplankton Spring bloom (Chlorophyll *a* content, grey line) and the associated bacterioplankton bloom (total cell count, black asterisks) off Helgoland, North Sea (54°11’03”N, 7°54’00”E) in 2018. Triangles indicate five sampling time points (Julian Days 102, 109, 115, 128, and 142).

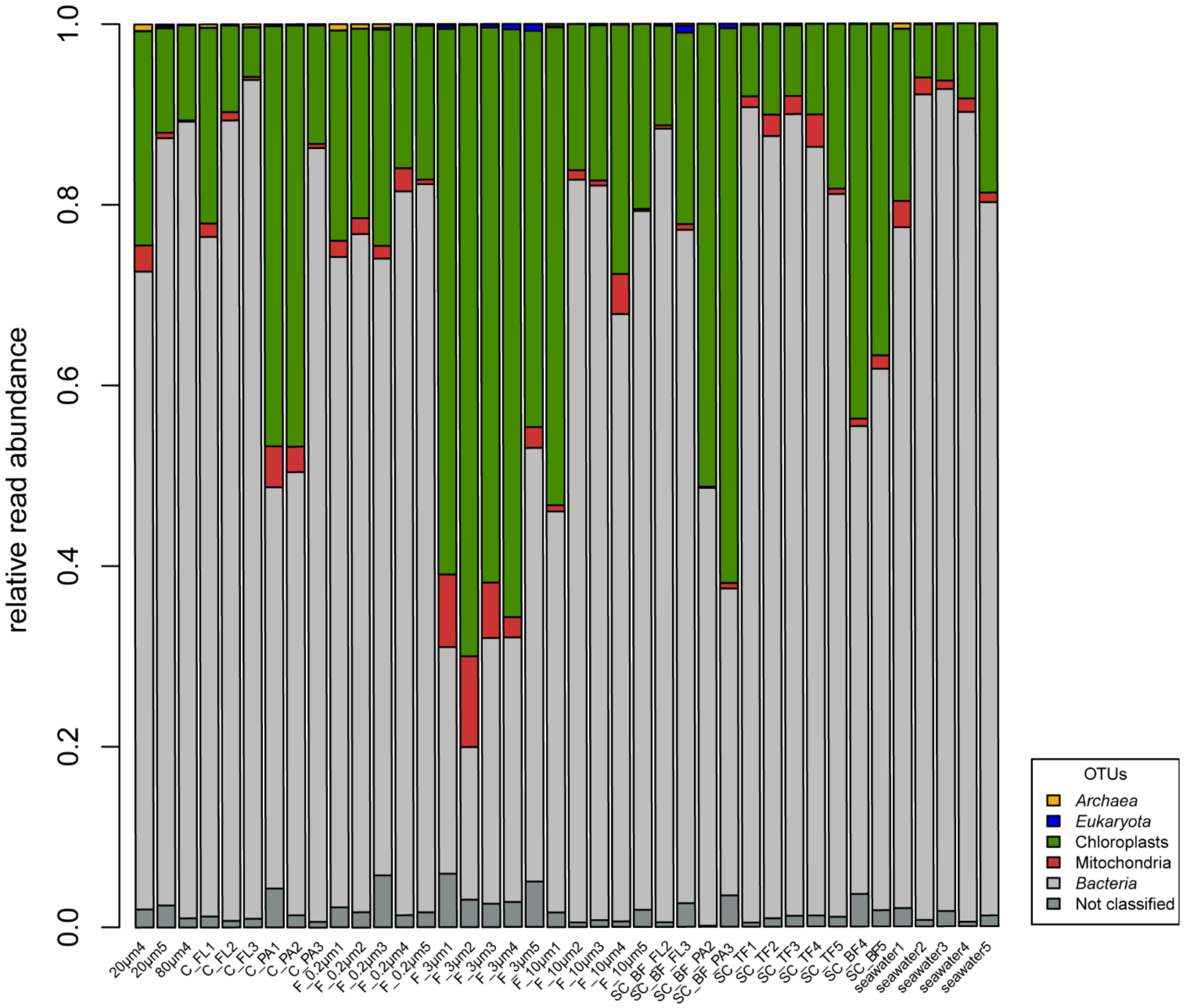

Supplementary Figure 2. Bar chart depicting the relative read abundances of *Archaea* (yellow), *Eukaryotes* (blue), chloroplasts (green), mitochondria (red), *Bacteria* (light grey) and operational taxonomic units (OTUs) with no close relatives (dark grey) in the Silva database (v. 132) in amplicon communities across different bacterioplankton separation techniques (x-axis) and sampling time points (number behind method, Julian Days 102, 109, 115, 128, and 142). *20µm*: 20µm plankton net fraction, *80µm*: 80 µm plankton net fraction, *C:* centrifugation, *F_0.2µm*: 3-0.2 µm filter fraction, *F_3µm*: 10-3 µm filter fraction, *F_10µm*: > 10 µm filter fraction, *FL*: free-living bacterial fraction, *SC_TF*: sedimentation cone top fraction, *SC_BF*: sedimentation cone bottom fraction. *SC_BF_FL/PA*: sedimentation cone bottom fraction separated by centrifugation into a supernatant (*FL*) and pellet (*PA*), *PA*: particle fraction.

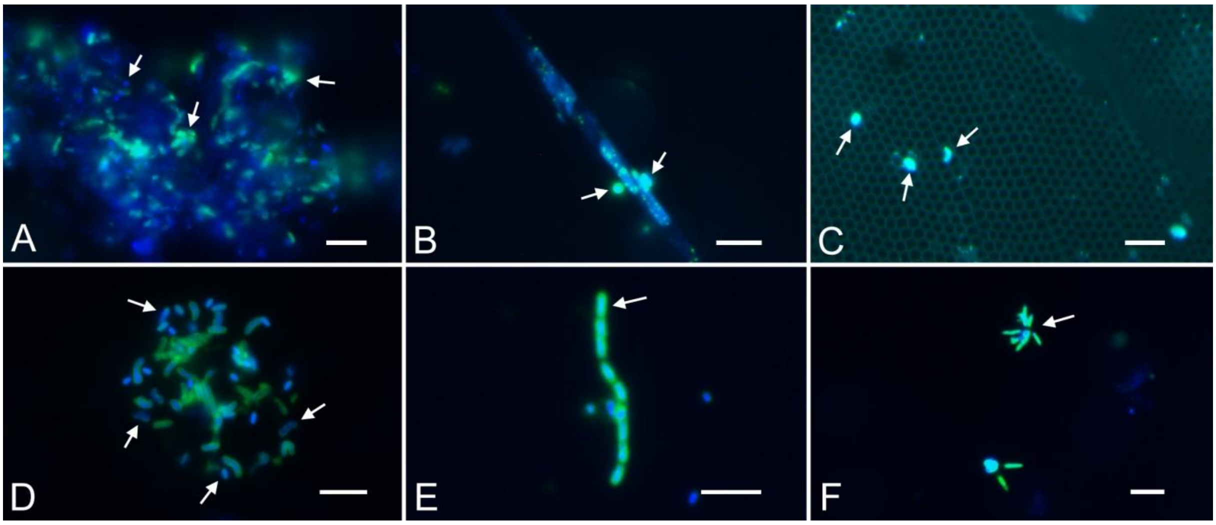

Supplementary Figure 3. Distributions of stained cells (blue: DAPI, green: EUBI-III probe and Alexa 488) in respect to particulate substrate **(A-C)** or other cells **(D-F)**. Samples were taken during a phytoplankton Spring bloom in 2018 off Helgoland (North Sea). **(A)** Cells (white arrows) in direct contact with a transparent exopolymeric particle, **(B)** an algae, or **(C)** a diatom shell; **(D)** Cell consortia with bacteria clustered in closer proximity to each other compared to the surrounding cell density, but without consistent contact points; **(E)** Filamentous cells; **(F)** Cells aggregated with a central distinctive connection point. Scale bars 5 µm.

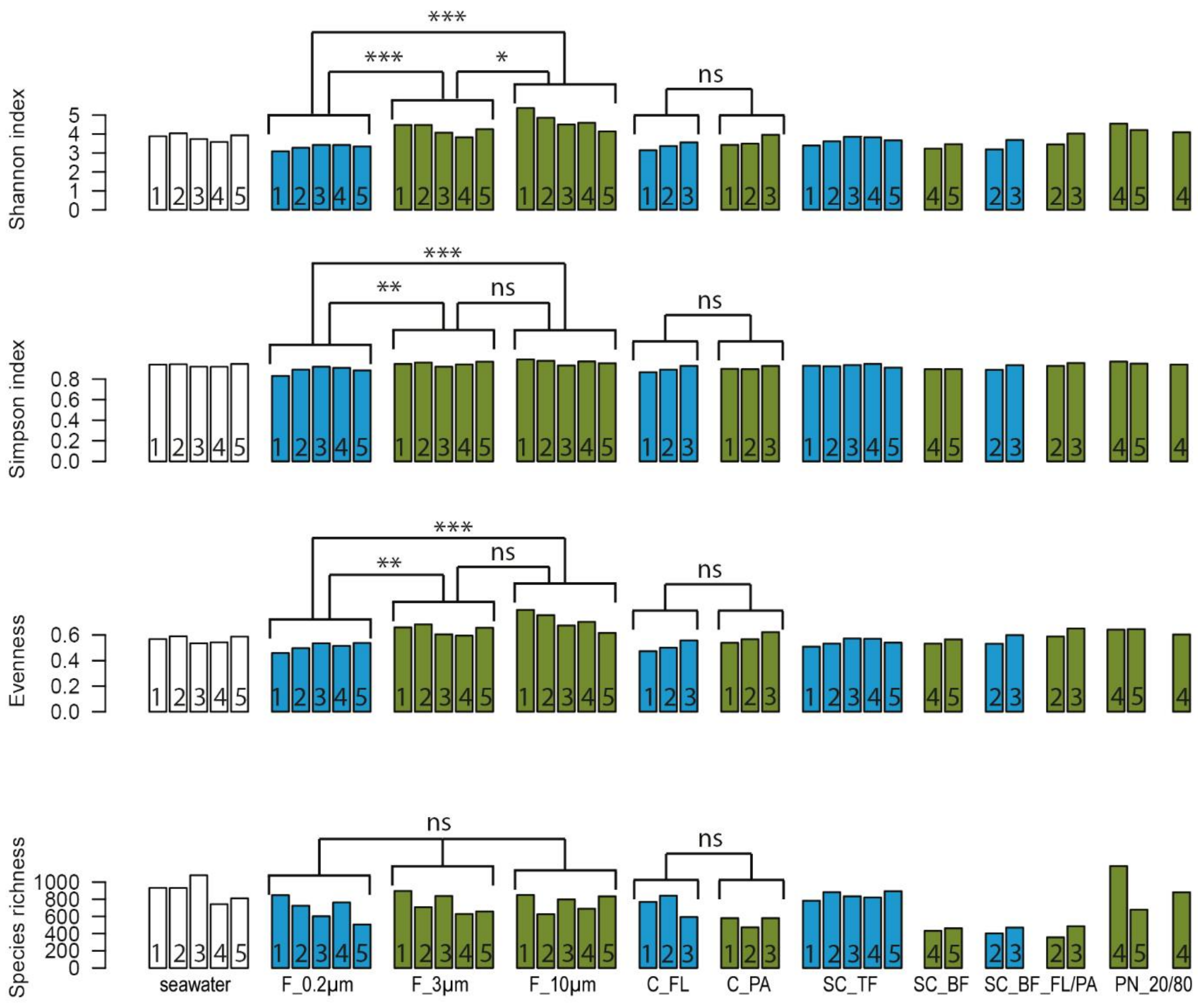

Supplementary Figure 4. Bar chart depicting diversity indices, species richness, and evenness of seawater and fractionated seawater samples taken during a phytoplankton Spring bloom off Helgoland (North Sea) in 2018. Seawater was either fractionated by size using sequential filtration with 10, 3, and 0.2 µm pore-sized filters (F_10µm/ 3µm/ 0.2µm) or plankton net catches with 20 or 80 µm pore size (PN_20/80); or they were split gravitationally by centrifugation (C_FL/ PA), with sedimentation cones (SC_TF/BF) or a combination of natural sedimentation in cones, followed by secondary differentiation by centrifugation (SC_BF_FL/PA). Numbers in the bars indicate the sampling times (Julian Days 102, 109, 115, 128, and 142). Brackets depict results of ANOVA and pairwise comparisons: *** $P < 0.0001$, ** $P < 0.001$, *$P < 0.05$, *ns:* no significant difference. *White* unfractionated seawater, *blue* free-living fraction, *green* particle fraction.

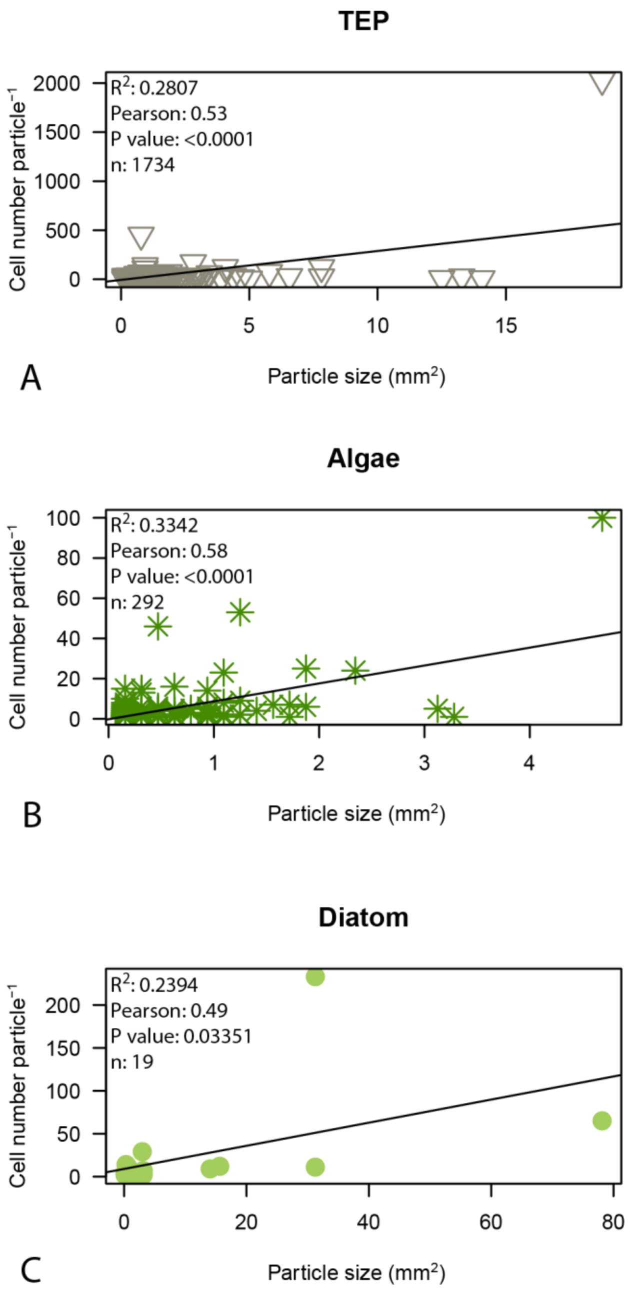

Supplementary Figure 5. Scatterplot displaying the relationship between cell number and particle size of particle-attached bacteria sampled during a phytoplankton Spring bloom off Helgoland (North Sea) in 2018. Bacteria attached to transparent exopolymeric particles **(A)**, algae **(B)**, or diatom debris **(C)**. The black line and R^2 value indicate the linear fit and its quality, respectively. *Pearson* best possible fit, *P value* significance, *n* number of samples.

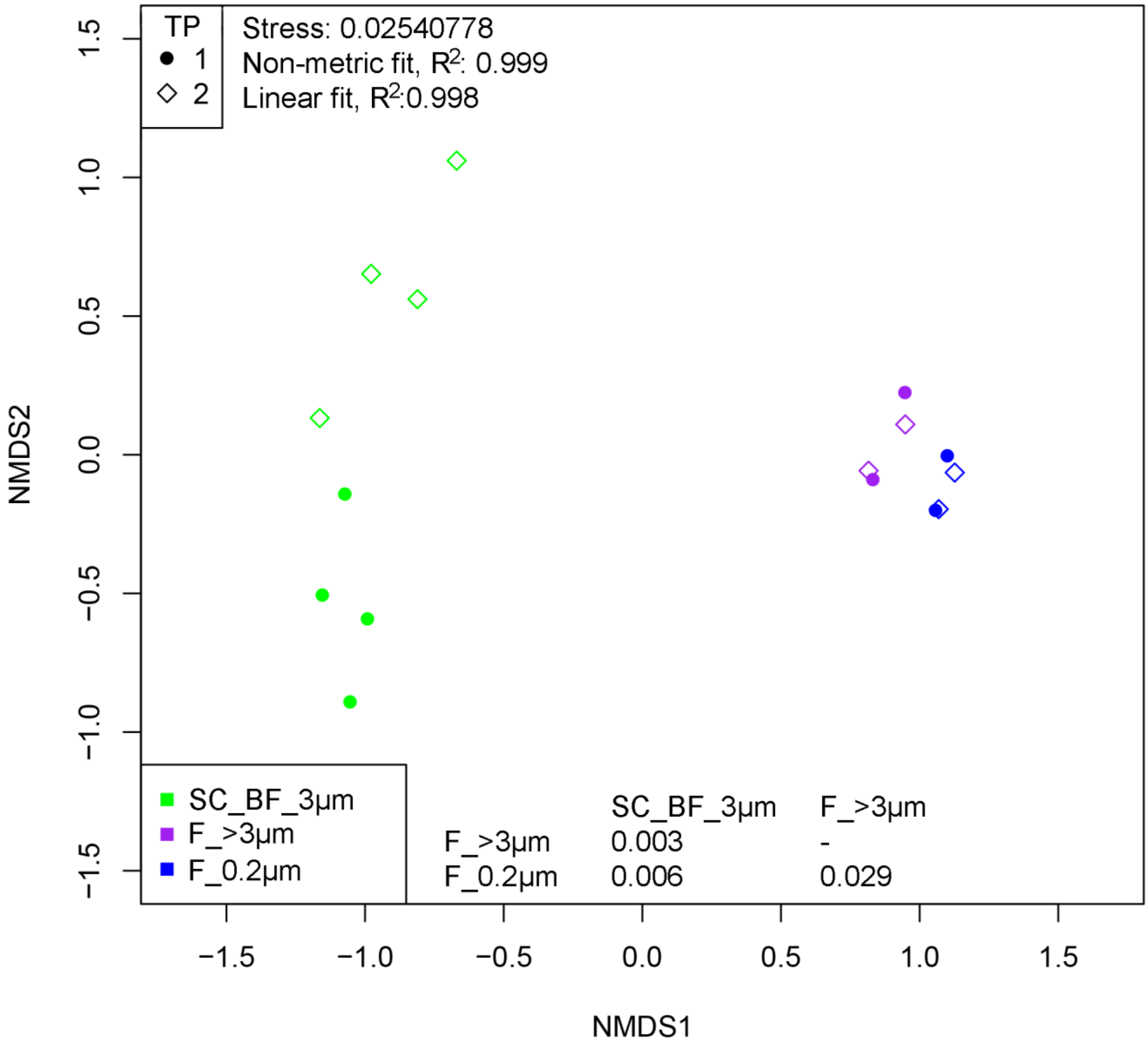

Supplementary Figure 6. Non-metric multidimensional scaling (NMDS) plot showing Bray Curtis dissimilarity of bacterial communities sampled during a phytoplankton Spring bloom off Helgoland (North Sea) in 2017. One liter of seawater was either separated by sequential filtration through a 3 µm (F_>3µm, purple) followed by a 0.2 µm pore sized filter (F_0.2µm, blue) directly after sampling, or it was transferred into a sedimentation cone, where it remained for 24 h until 5 mL of the bottom fraction were collected through a stopcock and filtered through a 3 µm pore sized filter (SC_BF_3µm, green). Symbols indicate the two sampling times (TP) Julian day 88 (circle) and 94 (diamond). Distance matrix (bottom of the plot) depicts the significance values (P value) based on a pairwise permutation MANOVA.
